# Self‐Cooperative Prodrug Nanovesicles Migrate Immune Evasion to Potentiate Chemoradiotherapy in Head and Neck Cancer

**DOI:** 10.1002/advs.202203263

**Published:** 2022-11-07

**Authors:** Yun Zhu, Shunan Zhang, Yi Lai, Jiaxing Pan, Fangmin Chen, Tingting Wang, Fengyang Wang, Zhiai Xu, Wenjun Yang, Haijun Yu

**Affiliations:** ^1^ Department of Oral and Maxillofacial‐Head and Neck Oncology Ninth People's Hospital College of Stomatology Shanghai Jiao Tong University School of Medicine; National Clinical Research Center for Oral Diseases Shanghai Key Laboratory of Stomatology Shanghai 200011 China; ^2^ Center of Pharmaceutics Shanghai Institute of Materia Medica Chinese Academy of Sciences Shanghai 201203 China; ^3^ School of Chemistry and Molecular Engineering East China Normal University Shanghai 200241 China; ^4^ Department of Gastroenterology Huadong Hospital Shanghai Medical College Fudan University Shanghai 200040 China; ^5^ Department of Medical Ultrasound Shanghai Tenth People's Hospital; Tongji University Shanghai 200072 China

**Keywords:** chemoradiotherapy, cooperative therapy, head and neck cancer, immune evasion, immunogenic cell death

## Abstract

Chemoradiotherapy is the standard of care for the clinical treatment of locally advanced head and neck cancers. However, the combination of ion radiation with free chemotherapeutics yields unsatisfactory therapeutic output and severe side effects due to the nonspecific biodistribution of the anticancer drugs. Herein, a self‐cooperative prodrug nanovesicle is reported for highly tumor‐specific chemoradiotherapy. The nanovesicles integrating a prodrug of oxaliplatin (OXA) can passively accumulate at the tumor site and penetrate deep into the tumor mass via matrix metalloproteinase 2‐mediated cleavage of the polyethylene glycol corona. The OXA prodrug can be restored inside the tumor cells with endogenous glutathione to trigger immunogenic cell death (ICD) of the tumor cells and sensitize the tumor to ion radiation. The nanovesicles can be further loaded with the JAK inhibitor ruxolitinib to abolish chemoradiotherapy‐induced programmed death ligand 1 (PD‐L1) upregulation on the surface of the tumor cells, thereby prompting chemoradiotherapy‐induced immunotherapy by blocking the interferon gamma‐Janus kinase‐signal transducer and activator of transcription axis. The prodrug nanoplatform reported herein might present a novel strategy to cooperatively enhance chemoradiotherapy of head and cancer and overcome PD‐L1‐dependent immune evasion.

## Graphical abstract

A prodrug nanovesicle integrating oxaliplatin prodrug and JAK inhibitor ruxolitinib is engineered for self‐cooperative chemoradiotherapy of head and neck cancer. The nanovesicles trigger immunogenic cell death of the tumor cells for sensitizing chemoradiotherapy and abolish programmed death ligand 1 expression on the surface of the tumor cells, thereby promoting combinatory chemoradiotherapy of cancer.

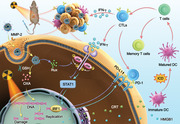

## 1 Introduction

Head and neck cancer is the sixth most frequent malignant tumor worldwide, and over 90% of head and neck cancers arising in the oral cavity or pharynx are head and neck squamous cell carcinomas (HNSCCs).^[^ ^1^ ^]^ Current practice guidelines recommend definitive concurrent chemotherapy and radiotherapy (chemoradiotherapy) for patients with localized advanced HNSCC.^[^ ^2^, ^3^, ^4^, ^5^ ^]^ Radiation can induce DNA damage in tumor cells. In contrast, an anticancer drug such as oxaliplatin (OXA) forms DNA cross‐links, which can disrupt DNA replication, enhancing the effects of radiotherapy.^[^ ^6^, ^7^ ^]^ Despite the fact that chemoradiotherapy is the standard of care for curative treatment of locally advanced HNSCC, chemoradiotherapy yields a 5‐year survival of less than 50% in HNSCC patients.^[^ ^8^, ^9^ ^]^ The poor prognosis of head and neck cancer patients could be attributed to a lack of tumor specificity, poor pharmacokinetics, and severe side toxicity of free chemotherapeutics. It therefore remains a formidable challenge to develop novel strategies for HNSCC therapy.

In recent years, immunotherapy, in particular immune checkpoint blockade (ICB) therapy, has emerged as a primary therapy modality for treating various solid tumors, including renal cell cancer, non‐small‐cell lung cancer, melanoma, and HNSCC.^[^ ^10^, ^11^ ^]^ Immune checkpoint inhibitors, including pembrolizumab and nivolumab, have demonstrated durable tumor remission in clinical therapy of HNSCC, but only 20% of HNSCC patients benefit from current ICB therapy.^[^ ^12^, ^13^, ^14^ ^]^ HNSCCs have low immunogenicity and insufficient intratumoral infiltration of CD8^+^ cytotoxic T lymphocytes (CTLs), which leads to unsatisfactory therapeutic outcomes of ICB therapy.^[^ ^15^, ^16^, ^17^ ^]^ It has been well documented that ion radiotherapy or chemotherapy with certain kinds of anticancer drugs (e.g., OXA and doxorubicin) can induce immunogenic cell death (ICD) of tumor cells to elicit immunogenicity and induce a systemic immune response to eradicate tumor residues and prevent tumor relapse.^[^ ^18^, ^19^, ^20^ ^]^ The tumor cells undergoing ICD exposure calreticulin (CRT) on the cell membrane surface act as an “eat‐me” signal and release high mobility group box 1 (HMGB1) as a “danger” signal to attract antigen‐presenting cells (APCs).^[^ ^21^, ^22^, ^23^ ^]^ The ICD effects promote dendritic cell (DC) maturation and recruit CTLs to tumor sites. However, the proinflammatory cytokine interferon gamma (IFN‐*γ*) secreted by tumor‐infiltrating CTLs upregulates negative immune regulators.^[^ ^24^ ^]^ For example, IFN‐*γ* induces programmed death ligand 1 (PD‐L1) expression on the tumor cell surface via the Janus kinase‐signal transducer and activator of transcription (JAK‐STAT) signaling pathway.^[^ ^25^, ^26^, ^27^ ^]^ PD‐L1 disables CTLs by binding programmed death receptor 1 on their surface and induces adaptive immune evasion.^[^ ^28^, ^29^ ^]^

Rational engineering of the intelligent nanovectors is currently one of the key issues for highly efficient drug delivery. To improve the performance of radiotherapy, X‐ray‐responsive chemotherapeutics can be integrated into the nanoplatform to synergistically improve the efficacy of radiotherapy with reduced side effects.^[^ ^30^ ^]^ Furthermore, the nanomaterials can also be formulated to deliver immunotherapeutics to enhance the antitumor efficacy of radio‐immunotherapy via recruiting CTLs and relieving the immunosuppressive tumor microenvironment.^[^ ^31^ ^]^

**Scheme 1:**
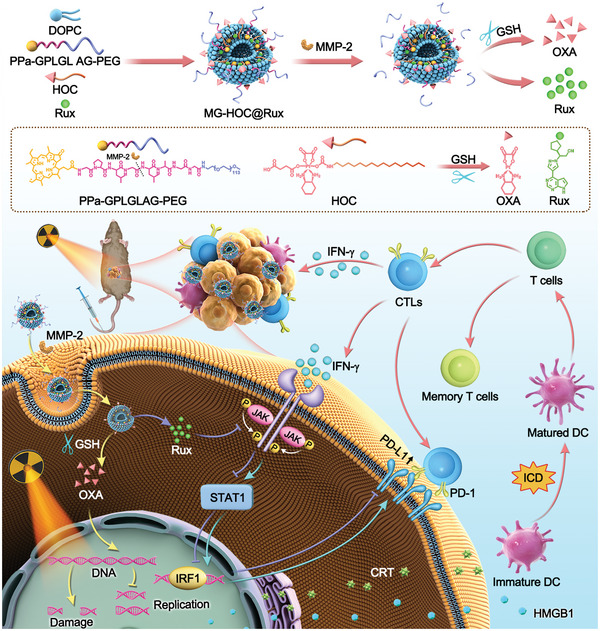
a) Self‐assembly of cooperative nanovesicles integrating MMP‐2‐sheddabke PEG corona, GSH‐activatable OXA prodrug, and JAKi Rux; b) Proposed mechanisms of cooperative nanovesicles‐based chemoradiotherapy of head and neck tumors by eliciting immunogenicity and overcoming adaptive immune resistance. The prodrug nanovesicles accumulated at the tumor site via MMP‐2‐mediated cleavage of the PEG corona. OXA and X‐rays initiate ICD in tumor cells, promote DC maturation, and activate CTLs for tumor regression. Additionally, Rux downregulates IFN‐*γ*‐inducible PD‐L1 expression on the surface of tumor cells to combat adaptive immune evasion.

To achieve precise chemoradiotherapy of HNSCC, we first engineered a tumor enzymatic microenvironment‐activatable nanovesicle for tumor‐specific delivery of OXA prodrug and radiotherapy sensitization. The nanovesicle comprised a matrix metalloproteinase 2 (MMP‐2)‐sheddable polyethylene glycol (PEG) corona to improve tumor‐specific accumulation and deep tumor penetration. The OXA prodrug is kept inert during blood circulation while being restored inside tumor cells with endogenous glutathione (GSH). We demonstrated that chemoradiotherapy with the OXA prodrug nanovesicle efficiently regressed HNSCC tumor growth and elicited ICD of the tumor cells. To maximize the ICD‐induced antitumor immunogenicity of chemoradiotherapy, we hypothesized that a combination of Janus kinase inhibitors (JAKi) with OXA prodrug nanovesicle‐based chemoradiotherapy might provoke systemic antitumor immunity while overcoming IFN‐*γ*‐induced immune evasion. Thus, the JAKi ruxolitinib (Rux) was integrated into the prodrug nanovesicles for potentiating chemoradiotherapy and immunotherapy. Once internalized into tumor cells, Rux can be released inside the tumor cells to inactivate the IFN‐*γ*‐JAK pathway and abolish PD‐L1 expression on the surface of tumor cells, thereby amplifying the immune response of prodrug nanovesicle‐based chemoimmunotherapy (**Scheme** **1**). To the best of our knowledge, this is the first study demonstrating the cooperative therapy effect between JAKi and chemoradiotherapy. Therefore, this study might provide a novel strategy for head and neck squamous cell carcinoma treatment.

## 2 Results and Discussion

### 2.1 Bioinformatics Analysis of MMP‐2 Expression and Characteristics of OXA Prodrug Nanovesicles

**Figure 1:**
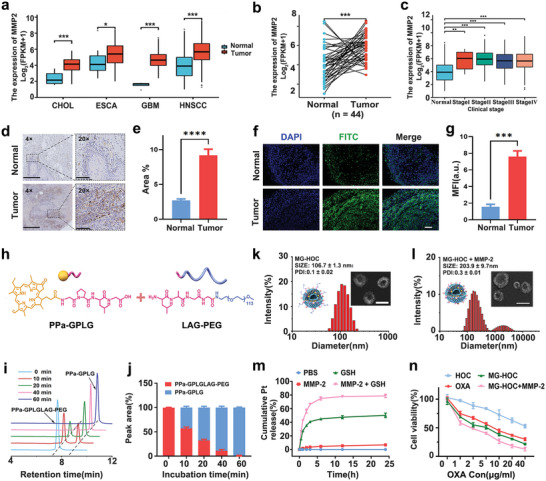
Bioinformatics analysis of MMP‐2 expression in human cancer patients by TCGA datasets and characteristics of MMP‐2‐sheddable OXA prodrug nanovesicles. a) Comparison of MMP‐2 expression in a broad variety of human cancers (CHOL: cholangiocarcinoma; ESCA: esophageal carcinoma; GBM: glioblastoma multiforme; HNSC: head and neck squamous cell carcinoma; Normal: normal tissues adjacent to the tumor; b) comparison of MMP‐2 expression in HNSCC tumors (*n* = 44) and paired normal tissues (*n* = 44); c) comparison of MMP‐2 expression in the different clinical stages of HNSCC; d,e) IHC examination of MMP‐2 expression in HNSCC tumors and normal tissues ex vivo (4 ×, scale bar = 625 µm; 20 ×, scale bar = 100 µm, *n* = 5); f,g) immunofluorescence examination of MMP‐2 expression in SCC7 tumors and normal tissues ex vivo (scale bar = 100 µm, *n* = 3); h) illustration of MMP‐2‐mediated cleavage of PPa‐GPLGLAG‐PEG conjugate; i) HPLC plots of PPa‐GPLGLAG‐PEG solution (1.0 mg mL^−1^) was incubated with 200 µg mL^−1^ of MMP‐2 at 37 °C; j) The quantitative ratio of PPa‐GPLGLAG‐PEG and PPa‐GPLG cleaved with MMP‐2; k, l) Particle size distribution collected via DLS. Insert: TEM images of MG‐HOC before and after 24 h of incubation with 200 µg mL^−1^ MMP‐2 (k, scale bar = 100 µm; j, scale bar = 200 µm); m) Pt release profile from MG‐HOC nanovesicles induced by GSH (10 mM) and MMP‐2 (200 µg mL^−1^); *n*) Cell viability of SCC7 cells after incubation with different concentrations of OXA, HOC, MG‐HOC, and MG‐HOC incubated with MMP‐2. The data are shown as the mean ± SD. *p < 0.05; **p < 0.01; ***p < 0.001; ****p < 0.0001.

To validate the principle for design of the MMP‐2‐sheddable OXA prodrug nanovesicles, tumor‐specific expression of MMP‐2 was investigated in 4 types of malignancies by The Cancer Genome Atlas (TCGA) TIMER database analysis, which showed that MMP‐2 is overexpressed in various human tumors (**Figure** **1**a), particularly in the tumor specimens of HNSCC tumors (*n* = 44) versus the paired normal tissue (*n* = 44) (Figure 1b). MMP‐2 is overexpressed in HNSCC tumor tissue at different clinical stages (Figure 1c), indicating the potential of MMP‐2 as an ideal stimulus to trigger tumor‐specific drug delivery.^[^ ^32^ ^]^ Moreover, we performed immunohistochemical (IHC) staining on human HNSCC tumors and immunofluorescence staining on mouse SCC7 HNSCC tumors. Semiquantitative data demonstrated that MMP‐2 expression in tumor tissues was significantly higher than that in normal tissues (Figure 1d‐g), further validating the potential of MMP‐2 as a tumor‐specific biomarker for tumor‐targeted drug delivery.

To achieve tumor‐specific OXA delivery and precise chemoradiotherapy in vivo, we engineered an MMP‐2‐sheddable nanovesicle by integrating an MMP‐2‐cleavable PEG corona, the fluorescence dye pheophorbide A (PPa) and a prodrug of OXA into a single nanoplatform. PPa was first conjugated with methoxy PEG amine (mPEG‐NH_2_) via the MMP‐2‐liable GPLGLAG peptide spacer GPLGLAG to obtain PPa‐GALGLPG‐PEG (namely, P‐M‐P) (Figure S1, Supporting Information). The MMP‐2 nonresponsive analog of P‐M‐P was synthesized by directly conjugating PPa and PEG‐NH_2_ via an imide bond (termed P‐P) (Figure S2, Supporting Information). Successful synthesis of P‐M‐P and P‐P was confirmed by proton nuclear magnetic resonance spectra and matrix‐assisted desorption/ionization‐time of flight mass spectrometry (Figures S3–S7, Supporting Information). High‐performance liquid chromatography (HPLC) analysis revealed that over 90% of P‐M‐P was degraded at the Leu‐Gly site after 60 min of incubation with 200 µg mL^−1^ MMP‐2, verifying the superior MMP‐2 sensitivity of the GALGLPG heptapeptide (Figure 1h‐j).

A lipophilic OXA prodrug of hexadecyl oxaliplatin carboxylic acid (HOC), which was inert during blood circulation to avoid drug leakage and converted to OXA under the endogenous reductive environment induced by excess GSH in tumor cells, was synthesized according to previous reports (Figure S8, Supporting Information).^[^ ^33^ ^]^The successful synthesis of HOC and the intermediate product was validated by ^1^H NMR spectra and electrospray ionization‐mass spectrometry spectra (Figures S9–S11, Supporting Information), respectively.

Subsequently, we constructed MMP‐2‐liable prodrug vesicles (MG‐HOCs) by film hydration and stepwise extrusion at a 1,2‐dioleoyl‐sn‐glycero‐3‐phosphoch‐olin (DOPC): PPa–GPLGLAG–PEG: HOC molar ratio of 76.3/3.3/12.5. Dynamic light scattering (DLS) examination revealed an average hydrodynamic diameter of 106.7 ± 1.3 nm and a narrow polydispersity index (PDI, e.g., 0.10 ± 0.02) of MG‐HOC (Figure 1k). The particle size and PDI of MG‐HOC increased upon 60 min of incubation in a buffer solution of MMP‐2. Transmission electron microscopic (TEM) images showed that MG‐HOC had a spherical morphology with a uniform size distribution, which agreed well with the DLS results. In contrast, the size distribution of MG‐HOC became heterogeneous after incubation with MMP‐2(Figure 1l), probably due to cleavage of the PEG corona and membrane fusion between MG‐HOC. Then, the particle size distribution of MMP‐2‐liable prodrug vesicles (MG‐HOCs) after MMP‐2 and GSH incubation was examined. TEM examination displayed that the particle size and size distribution of the MG‐HOC nanovesicles dramatically increased upon 24 h incubation with 200 µg mL^−1^ of MMP‐2 and 10 × 10^−3^ m of GSH (Figure S12, Supporting Information), suggesting GSH incubation promoted OXA release and disintegrates the nanovesicles.

The reduction sensitivity of the prodrug nanovesicles was examined by investigating the Pt release profile with an inductively coupled plasma mass spectrometer . MG‐HOC nanovesicles displayed negligible OXA release upon 37 °C water incubation. In contrast, 50.2 ± 3.3% of OXA was released from MG‐HOC after 24 h of incubation with 10 × 10^−3^ m GSH alone (Figure 1m). Remarkably, up to 78.8 ± 2.5% of OXA was released from MG‐HOC nanovesicles upon 24 h incubation with both MMP‐2 and GSH due to increased access of GSH to HOC via MMP‐2‐mediated cleavage of the PEG corona. Then, the effect of MG‐HOC on the viability of SCC7 cells was assessed by Cell Counting Kit‐8 (CCK8) cell viability assay. HOC displayed a minor cytotoxic effect compared with free OXA, while MG‐HOC incubated with MMP‐2 showed an apparent cytotoxic effect on tumor cells (Figure 1n).

### 2.2 Tumor Penetration of the Nanovesicles In Vitro and Biodistribution In Vivo

**Figure 2:**
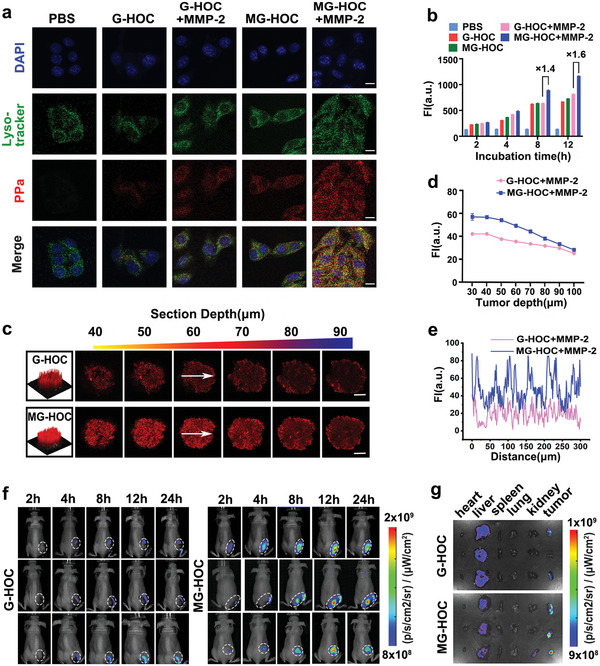
Biodistribution of MG‐HOC nanovesicles in an SCC7 tumor‐bearing mouse model in v. a) CLSM and b) flow cytometric analysis of intracellular uptake of MG‐HOC in SCC7 cells in vitro at a PPa concentration of 2.0 × 10^−6^ m. The prodrug vesicles were pretreated with 200 µg mL^−1^ MMP‐2 for 1 h at 37 °C. CLSM analysis was performed 12 h post‐nanoparticle incubation (scale bar = 20 µm); c) CLSM analysis of the MG‐HOC and G‐HOC distribution in MCSs of SCC7 cells (scale bar = 100 µm) and 2.5‐D heat map; d) fluorescence intensity profile of the MCS's central region versus Z‐axis depth after 12 h incubation with G‐HOC or MG‐HOC; e) Fluorescence intensity profile of the MCS along the arrow region; f) IVIS images of the MG‐HOC and G‐HOC distribution in SCC7 tumor‐bearing mice in vivo; g) IVIS images of the MG‐HOC and G‐HOC distribution in the major organs of tumor‐bearing mice ex vivo as examined 24 h postinjection.

To demonstrate the advantage of MMP‐2‐sheddable nanovesicles, we investigated the intracellular uptake behavior of MG‐HOC prodrug nanovesicles in murine SCC7 tumor cells in vitro. Confocal laser scanning microscopy (CLSM) examination showed that MMP‐2 treatment dramatically facilitated the intracellular uptake of MG‐HOC (**Figure** **2**a). The prodrug nanovesicles were taken up through the endocytosis pathway. Flow cytometry analysis further identified 1.6‐fold higher cellular uptake of MMP‐2‐pretreated MG‐HOC than that of the MMP‐2 nonresponsive G‐HOC group, verifying that cleavage of the PEG corona promoted cellular uptake of the prodrug nanovesicles (Figure 2b).

Given the increased intracellular uptake of the prodrug nanovesicles in vitro via MMP‐2‐mediated PEG cleavage, we then evaluated the tumor penetration ability of MG‐HOC in a three‐dimensional multicellular spheroid (MCS) tumor model in vitro. Figure 2c shows that MMP‐2‐nonresponsive G‐HOC nanovesicles were dominantly entrapped in the peripheral area of the MCS. In contrast, MMP‐2‐responsive MG‐HOC nanovesicles penetrated the interior zone in sharp contrast after 24 h of incubation. For instance, MG‐HOC nanovesicles displayed ≈1.44‐fold higher tumor penetration ability than G‐HOC nanovesicles at a section depth of 60 µm in the MCS, further verifying that the increased tumor penetration of MG‐HOC nanovesicles was attributed to MMP‐2‐sensitive cleavage of the PEG corona (Figure 2d,e).

To investigate tumor accumulation and penetration of the prodrug vesicles in vivo, biodistribution of the nanovesicles was examined in SCC7 tumor‐bearing nude mice in vivo (Figure 2f). MG‐HOC and G‐HOC nanovesicles were administered through intravenous (i.v.) injection at an identical PPa dose of 2.0 mg kg^−1^. MG‐HOC showed increased fluorescence emission of PPa at the tumor site and peaked at 12 h postinjection. In contrast, the MG‐HOC group displayed higher fluorescence intensity at the tumor site than the G‐HOC group at 12 and 24 h postinjection (Figure S13a, Supporting Information). Ex vivo fluorescence imaging of the tumor tissues and quantification of the tumoral fluorescence signal confirmed higher tumor accumulation of MG‐HOC than G‐HOC (Figure 2g and Figure S13b, Supporting Information). Moreover, MG‐HOC highly efficiently penetrated into the deep tumor, as verified by an immunofluorescence assay of the tumor sections (Figure S14, Supporting Information), which provided more evidence for PEG cleavage‐induced intratumoral accumulation and retention of the sheddable nanovesicles. These results implied the promising potential of MG‐HOC for tumor‐specific drug delivery in vitro and in vivo.

### 2.3 Radiosensitization and ICD Induction with the Prodrug Nanovesicles In Vitro

**Figure 3:**
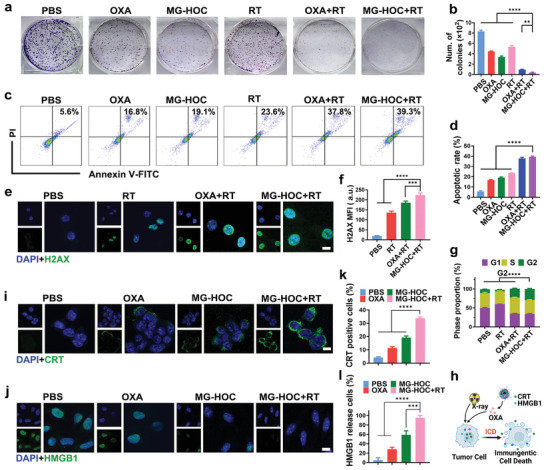
Radiosensitization and ICD of MG‐HOC in vitro. a,b) Colony formation ability of SCC7 cells inhibited with indicated treatments for 8 d; c,d) the apoptosis rate of SCC7 cells with indicated treatments; e,f) Representative H2AX immunofluorescence images with different treatments (scale bar = 10 µm), and semiquantitative analysis of intracellular fluorescence intensity; g) chemoradiotherapy induced cell cycle arrest in the G2 phase of SCC7 cells. The proportion of the G2 phase was significantly increased, while that of the G1 phase was decreased; h) schematic illustration of chemoradiotherapy‐induced ICD of tumor cells as characterized by CRT exposure and HMGB1 release (created with BioRender.com); i) chemoradiotherapy‐triggered CRT exposure and j) HMGB1 release by SCC7 cells after 24 h of incubation in vitro (scale bar = 10 µm); k) Flow cytometry examination of CRT exposure and l) fluorescence semiquantitative analysis of HMGB1 release in vitro. The data are shown as the mean ± SD. *p < 0.05; **p < 0.01; ***p < 0.001; ****p < 0.0001.

The ability of X‐ray to damage SCC7 cells was limited, and the cell viability after X‐ray treatment with a large dose of 8.0 Gy of radiation was ≈51.7 ± 2.2% (Figure S15, Supporting Information). Radiosensitization with the MG‐HOC nanovesicles was then exploited in SCC7 cells in vitro. A clonogenic survival assay indicated that ion radiation reduced cell survival to 64.4% of the PBS group. In contrast, ion irradiation of MG‐HOC‐treated SCC7 cells at 8.0 Gy potently decreased cell colony formation to 4.3% of the PBS group, indicating the promising potential of MG‐HOC nanovesicles as physical sensitizers for radiotherapy (**Figure** **3**a,b). Moreover, flow cytometry analysis further confirmed that MG‐HOC+RT treatment induced prominent cell apoptosis (Figure 3c,d).

Afterward, *γ*‐H2AX, a marker for DNA damage,^[^ ^34^ ^]^ was used to investigate the radiosensitization mechanism of the prodrug liposome. Cellular *γ*‐H2AX expression was examined in SCC7 cells by immunofluorescence staining. The fluorescence intensity of the *γ*‐H2AX antibody in MG‐HOC+RT was higher than that in the other groups (Figure 3e,f), suggesting that MG‐HOC could promote radiation damage to tumor cell DNA. Additionally, the proportion of the G2 phase was significantly increased in the MG‐HOC+RT group as determined by flow cytometry assay, suggesting that MG‐HOC could induce G2 phase arrest in SCC7 cells (Figure 3g and Figure S16, Supporting Information).

We next examined the effect of chemoradiotherapy‐induced ICD by measuring CRT expression and HMGB1 release (Figure 3h). CLSM examination showed that MG‐HOC+RT induced apparent CRT exposure on the membrane and HMGB1 release from the nucleus of treated tumor cells (Figure 3i,j). Moreover, flow cytometry analysis revealed that MG‐HOC+RT induced 3.0‐fold more CRT exposure than the OXA group and 1.8‐fold more than the MG‐HOC group (Figure 3k), and MG‐HOC+RT caused 3.4‐fold more HMGB1 release than the OXA group and 1.6‐fold more than the MG‐HOC group (Figure 3l).

DC play crucial roles in regulating innate and adaptive antitumor immunity,^[^ ^35^ ^]^ presenting tumor‐specific antigens to naive T cells to activate CTLs.^[^ ^36^ ^]^ MG‐HOC‐induced immunogenicity was thus investigated by analyzing tumor cell‐induced DC maturation. Freshly isolated bone marrow‐derived dendritic cells (BMDC) were incubated with SCC7 cells pretreated with prodrug nanovesicles. The fraction of mature DC (CD11c^+^CD80^+^CD86^+^) was then determined by flow cytometry analysis. MG‐HOC+RT induced 23.6 ± 3.5% BMDC maturation, which was 2.7‐fold more efficient than free OXA, indicating that MG‐HOC was able to elicit an immune response in vitro (Figure S17, Supporting Information).

### 2.4 Antitumor Performance of the Prodrug Nanovesicles In Vivo

**Figure 4:**
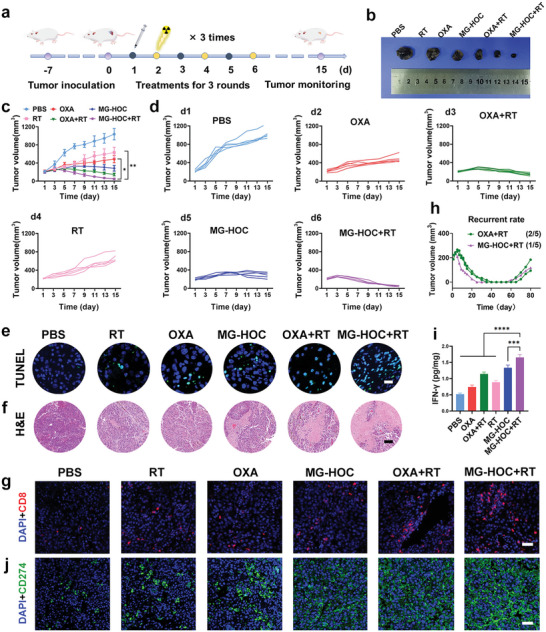
Theapeutic efficacy of MG‐HOC in vivo. a) Schematic illustration of the treatment strategy of MG‐HOC; b) The tumor photographs of SCC7 tumors after different treatments; c) averaged and d) individual tumor volumes of SCC7 tumor‐bearing mice upon the specified treatments (*n* = 5); e) TUNEL staining of SCC7 tumors at the end of treatments (scale bar = 20 µm); f) H&E staining of SCC7 tumor sections at the end of treatments (scale bar = 100 µm); g) immunofluorescence staining of CD8^+^ T lymphocytes in SCC7 tumors at day 5 post the final treatments (scale bar = 50 µm); h) tumor volumes of recurrent SCC7 tumor‐bearing mice after complete regression and recurrence rate of the groups; i) ELISA examination of intratumoral IFN‐*γ* secretion following different treatments and examined 5th‐day postinjection (*n* = 3); j) CLSM examination of CD274 expression in SCC7 tumor tissues ex vivo (scale bar = 50 µm). The data are shown as the mean ± SD. * *p* < 0.05; ** *p* < 0.01; *** *p* < 0.001; **** *p* < 0.0001.

Encouraged by the superior tumor accumulation and radiosensitization as well as ICD induction properties of MG‐HOC nanovesicles, we next evaluated their antitumor performance for combined chemoradiotherapy and immunotherapy in a subcutaneous SCC7 tumor model. The SCC7 tumor model was established by implanting 2 × 10^6^ cells into the subcutaneous flanks of each mouse. The mice were randomly divided into six groups (PBS, OXA, OXA+RT, RT, MG‐HOC, and MG‐HOC+RT) when the tumor volume reached 200 mm^3^ (*n* = 5) and then i.v. injected with PBS, OXA, or MG‐HOC at an OXA dose of 1.0 mg kg^−1^, followed by ion irradiation at a dose of 4.0 Gy. The treatments were performed three times every 2 d (**Figure** **4**a). Treatment with OXA or RT alone showed negligible tumor inhibition, while OXA+RT moderately inhibited the growth of SCC7 tumors. In contrast, MG‐HOC+RT dramatically regressed tumor growth (Figure 4b–d). Terminal deoxynucleotidyl transferase dUTP nick‐end labeling (TUNEL) and hematoxylin‐eosin (H&E) staining further revealed that MG‐HOC+RT remarkably induced apoptosis and necrosis of tumor cells in vivo (Figures 4e,f). CLSM examination of H2AX showed that MG‐HOC+RT caused apparent DNA damage in the tumor sections (Figure S18, Supporting Information).

Immunofluorescence staining of the tumor sections ex vivo showed that MG‐HOC +RT promoted intratumoral infiltration of CD8^+^ T cells compared with the other groups (Figure 4g, Figure S22, Supporting Information). CLSM examination showed that MG‐HOC+RT induced apparent CRT secretion on the membrane of tumor cells, suggesting that this treatment activates a strong ICD effect in vivo (Figure S19, Supporting Information). These findings implied good potential of combined MG‐HOC and RT to suppress SCC7 tumors. H&E staining of the tumor sections showed negligible histopathological changes in the major organs (e.g., heart, liver, spleen, lung and kidney) in all the treated animals, indicating good biosafety of the prodrug nanovesicles for clinical translation (Figure S20, Supporting Information). Negligible body weight change was found in SCC7 tumor‐bearing mice, indicating good biosafety of the prodrug nanovesicles (Figure S21, Supporting Information).

Despite the impressive antitumor performance of MG‐HOC nanovesicles, tumors relapsed from days 60–80 in mice receiving MG‐HOC+RT treatment (Figure 4h). To clarify the mechanisms underlying tumor recurrence, we investigated the intratumoral secretion of the proinflammatory cytokines IFN‐*γ* and PD‐L1 on tumor cells since upregulating PD‐L1 expression may cause immune evasion of some tumors. MG‐HOC+RT elicited intratumoral secretion of IFN‐*γ* 3.2‐fold higher than that in the PBS group and 2.2‐fold higher than that in the OXA group, indicating that chemoradiotherapy with the prodrug nanovesicles elicited a remarkable inflammatory response (Figure 4i). Moreover, MG‐HOC+RT treatment also increased PD‐L1 expression in tumor tissues (Figure 4j and Figure S23, Supporting Information), implying that IFN upregulates PD‐L1 expression on tumor cell surfaces. These data collectively verified that chemoradiotherapy can recruit intratumoral infiltration of CTLs but induce adaptive immune resistance by secreting IFN‐*γ* and triggering PD‐L1 upregulation on tumor cells.^[^ ^37^, ^38^ ^]^

### 2.5 Bioinformatics Analysis of TCGA HNSCC Datasets

**Figure 5:**
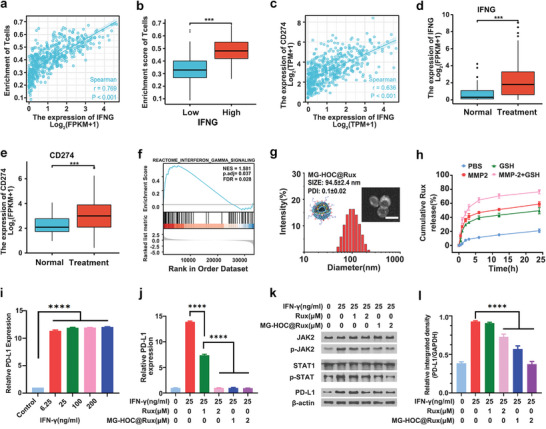
TCGA analysis of tumor‐infiltrating T lymphocytes and the IFNG pathway in HNSCC datasets and characteristics of JAKi‐loaded MG‐HOC@Rux nanovesicles. a) Correlation between the expression of IFNG and enrichment of T cells (*p* < 0.001); b) comparison of enrichment of T cells in the high expression of IFNG tumor and low expression tumor; c) correlation between the expression of IFNG and the expression of CD274 (*p* < 0.001); d,e) comparison of IFNG and CD274 mRNA expression in the treated HNSCC tumor and normal tissues; f) GO and KEGG pathways enriched in HNSCC samples with platinum‐based chemotherapy or radiotherapy with GSEA (P.adj < 0.05); g) particle size distribution collected via DLS. Insert: TEM images of MG‐HOC@Rux (scale bar = 100 µm); h) Rux release profile from MG‐HOC@Rux nanovesicles induced by GSH (10 mM) and MMP‐2 (200 µg mL^−1^); i) flow cytometry examination of IFN‐*γ*‐elicited PD‐L1 expression in SCC7 cells in vitro; j) flow cytometry examination of PD‐L1 expression in SCC7 tumor cells upon different treatments (24 h coincubation with free Rux or Rux nanoparticles and 25 ng mL^−1^ IFN‐*γ*) in vitro; k) western blot assay of PD‐L1 expression in SCC7 tumor cells upon different treatments (24 h coincubation with free Rux or Rux nanovesicles and 25 ng mL^−1^ IFN‐*γ*) in vitro; l) semiquantitative analysis of Western blot assay‐determined PD‐L1 expression in SCC7 cells by ImageJ software (*n* = 3). The data are shown as the mean ± SD. *p < 0.05; ** *p* < 0.01; *** *p* < 0.001; **** *p* < 0.0001.

To elucidate the mechanism underlying IFN‐*γ*‐induced PD‐L1 expression, microarray data and clinical information of HNSCC samples from TCGA were analyzed in HNSCC patients exposed to platinum‐based chemotherapy or radiotherapy. Differential expression analysis was conducted on the samples. Interferon gamma (IFNG) expression was significantly correlated with T‐cell infiltration (*p* < 0.01) (**Figure** **5**a). The high expression group of IFNG was more evident in T‐cell infiltration than the low expression group (Figure 5b). We also found that IFNG expression was strongly correlated with the expression of PD‐L1 (CD274) (*p* < 0.01), suggesting that IFNG might account for CD274 upregulation (Figure 5c).

Differential expression analysis was further conducted on 175 tumor specimens that received platinum‐based chemotherapy or radiotherapy within 502 tumor samples filtered by clinical treatment information. We found that the expression of IFNG and CD274 was significantly upregulated in samples that received platinum‐based chemotherapy and radiotherapy (Figure 5d,e).

To validate the immune induction function of chemoradiotherapy in HNSCC, Gene Set Enrichment Analysis was conducted to identify Gene Ontology and Kyoto Encyclopedia of Genes and Genomes pathways enriched in HNSCC samples with platinum‐based chemotherapy or radiotherapy. The “REACTOME Interferon gamma signaling” gene set showed statistical significance (Figure 5f), implying that IFN‐*γ* signaling in HNSCC was affected by radiotherapy or platinum‐based chemotherapy.

### 2.6 Characteristics of OXA Prodrug and JAKi co‐delivered Nanovesicles

To completely eradicate the tumor cells and prevent tumor recurrence caused by chemoradiotherapy‐induced PD‐L1 upregulation, the JAKi Rux was integrated into OXA prodrug nanovesicles (namely, MG‐HOC@Rux) for tumor‐specific blockade of the IFN‐*γ*‐JAK pathway and abolition of PD‐L1 expression. DLS analysis and TEM images showed that the particle size of MG‐HOC@Rux is slightly smaller than that of MG‐HOC, which may be attributed to the increased hydrophobic force after loading Rux. MG‐HOC@Rux displayed an average hydrodynamic diameter of 94.5 ± 2.4 nm and a PDI of 0.10 ± 0.02 (Figure 5g).

To elucidate the mechanism of Rux release from the nanovesicles, we investigated Rux release profile in the presence of MMP‐2 and GSH (Figure 5h). HPLC examination showed that without the addition of GSH and MMP‐2, less than 10% of Rux was released from the MG‐HOC@Rux nanovesicles upon 6 h incubation in PBS. Rux release rate increased up to ∼40% in the presence of 10 mM of GSH or 200 µg mL^−1^ of MMP‐2. Rux release ratio was further accelerated by incubating with both GSH and MMP‐2, which was ∼20% higher than that released in the MMP‐2 condition. DLS examination was employed to monitor particle size change in the presence of GSH and MMP‐2 (Figure S24, Supporting Information). The hydrodynamic diameter of MG‐HOC@Rux nanovesicles kept unchanged post 24 h incubation in 10 mM of GSH solution. However, ∼ 50% of Rux was released in 24 h since GSH could loosen the MG‐HOC@Rux nanovesicles and accelerate Rux release by reducing the HOC prodrug. Notably, the hydrodynamic diameter and size distribution of MG‐HOC@Rux nanovesicles became heterogeneous (PDI ∼ 0.5) upon 24 h incubation with MMP‐2. The hydrodynamic diameter of MG‐HOC@Rux increased up to 502.8 ± 15.5 nm with a broad PDI of 0.4 ± 0.06 post 24 h incubation with MMP‐2 and GSH. This phenomenon can be explained by MMP‐2‐mediated cleavage of PEG corona and membrane fusion between the nanovesicles, which could promote Rux release from the hydrophobic lipid shell.

To investigate whether Rux‐loaded nanovesicles relieved IFN‐*γ*‐inducible adaptive immune resistance in vitro, SCC7 tumor cells were first pretreated with IFN‐*γ* and then incubated with Rux‐loaded nanovesicles for 24 h. Flow cytometry analysis of PD‐L1 expression in SCC7 tumor cells in vitro revealed that IFN‐*γ* dramatically elicited PD‐L1 expression in tumor cells as a function of IFN‐*γ* concentration (Figure 5i and Figure S25, Supporting Information). In contrast, IFN‐*γ*‐inducible PD‐L1 expression was abolished by the nanovesicles (Figure 5j). Western blot assays further confirmed that MG‐HOC@Rux nanovesicles blocked IFN‐*γ*‐induced PD‐L1 upregulation (Figure 5k,l).

### 2.7 Immunoassay and Antitumor Performance of MG‐HOC@Rux Nanovesicles In Vivo

**Figure 6:**
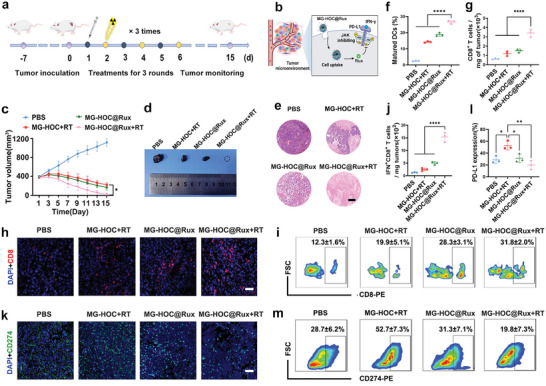
Antitumor effect and immune analysis in vivo. a) Experimental schedule of MG‐HOC@Rux nanovesicle‐based chemoradiotherapy in vivo; b) schematic illustration of Rux‐mediated suppression of PD‐L1 expression by blocking the JAK‐STAT pathway (created with BioRender.com); c) tumor growth curves of SCC7 tumor‐bearing mice upon treatments (*n* = 4); d) representative photographs of SCC7 tumors at the end of antitumor study; e) H&E staining of SCC7 tumors at the end of treatments (scale bar = 100 µm); f) DC maturation in the tumor‐draining LNs following different treatments and examined 5th day postinjection; g) tumor mass‐normalized tumor‐infiltrating CD8^+^ T cells; h) immunofluorescence staining of CD8^+^ T lymphocytes in SCC7 tumors examined at day‐5 post the final treatment (scale bar = 50 µm); i) flow cytometric plots of tumor‐infiltrating CD8^+^ T cell; j) tumor mass‐normalized number of IFN‐*γ* ^+^CD8^+^ effector T cells examined at day‐5 post‐treatment; k) immunofluorescence staining of PD‐L1 expression in SCC7 tumor‐bearing C3H mice receiving different treatments (scale bar = 50 µm); l,m) flow cytometric assay of PD‐L1‐expressing tumor cells (CD274^+^CD45^−^) in vivo (*n* = 3). The data are shown as the mean ± SD. *p < 0.05; ** *p* < 0.01; *** *p* < 0.001; **** *p* < 0.0001.

The antitumor efficacy of MG‐HOC@Rux+RT was then investigated in the SCC7 tumor‐bearing C3H mouse model in vivo (**Figure** **6**a). The treatment strategy of suppressing PD‐L1 in tumor cells by Rux release from MG‐HOC@Ru through the JAK‐STAT pathway is shown in Figure 6b. The mice were randomly divided into four groups (PBS, MG‐HOC+RT, MG‐HOC@Rux, and MG‐HOC@Rux+RT) when the tumor volume reached 400 mm^3^. The mouse groups then i.v. injected with PBS, MG‐HOC or MG‐HOC@Rux at an OXA dose of 1.0 mg kg^−1^, followed by ion radiation at an identical dose of 4.0 Gy. MG‐HOC@Rux+RT remarkably regressed SCC7 tumor growth and even eradicated the tumor xenografts (Figure 6c,d), implying cooperative therapeutic performance between chemoradiotherapy and JAK inhibition. H&E staining further revealed obvious necrosis of tumors in the MG‐HOC@Rux+RT group (Figures 6e and Scheme 1).

To exploit the mechanism underlying the superior antitumor performance of MG‐HOC@Rux, DC maturation in the tumor‐draining LNs and tumor‐infiltrating T lymphocytes was examined at the end of the antitumor study. The MG‐HOC@Rux+RT group remarkably elicited DC maturation, verifying that the treatment efficiently elicited an immune response by inducing ICD in the tumor cells in vivo (Figure 6f and Figure S26, Supporting Information). Treatment with MG‐HOC@Rux+RT markedly promoted intratumoral infiltration of CD8^+^ T cells compared with other treatments (Figure 6g), as validated by the following immunofluorescence staining of CD8^+^ T cells in the tumor sections (Figure 6h,i). Apart from increased intratumoral infiltration of CD8^+^ T cells, combinatory treatment with MG‐HOC@Rux+RT significantly increased the percentage of effector T lymphocytes (IFN‐*γ* ^+^CD8^+^ T cells) (Figure 6j and Figure S27, Supporting Information). All these data indicate that chemoradiotherapy with MG‐HOC@Rux nanovesicles dramatically activated the protective immune response and relieved the immunosuppressive tumor microenvironment.

The immune memory effects are crucial for long‐term tumor regression. We thus examined the memory T cells (CD3^+^CD8^+^CD44^+^CD127^+^) in the spleen at the end of the antitumor study. The fraction of memory T cells in the MG‐HOC@Rux+RT group was obviously higher than the other groups (Figure S28, Supporting Information), suggesting chemoimmunotherapy based on MG‐HOC@Rux+RT treatment induced a long‐term immune memory to prevent tumor recurrent.

Immunofluorescence staining revealed that MG‐HOC@Rux+RT negligibly elicited PD‐L1 expression in the tumor, in significant contrast to MG‐HOC+RT (Figure 6k). For instance, PD‐L1^+^ tumor cells in the MG‐HOC@Rux+RT group were 1.7‐fold lower than those in the MG‐HOC+RT group because Rux abolished PD‐L1 expression (Figure 6l,m). Additionally, H&E staining showed negligible histopathological changes in the major organs in all treated groups (Figure S29, Supporting Information), and there were no noticeable body weight changes in SCC7 tumor‐bearing mice, indicating excellent biosafety for clinical translation (Figure S30, Supporting Information). Overall, our data showed that the Rux‐loaded nanovesicles blocked IFN‐*γ*‐inducible PD‐L1 upregulation and improved chemoimmunotherapy. Despite JAKi was reported to inhibit chemokine secretion in vivo.^[^ ^39^, ^40^ ^]^ JAKi might thus impair chemokine‐mediated T lymphocyte migration. On the other hand, iradiation can induce intratumoral section of the proinflammatory chemotactic factors to recruit antitumor effector T cells.^[^ ^41^ ^]^ Therefore, the combination of ionradiation with JAKi was employed to elicit antitumor immunogenicity and inhibit JAK pathway‐induced PD‐L1 expression.

## 3 Conclusion

We have presented an OXA prodrug and JAKi Rux coloaded nanovesicle for chemoradiotherapy of head and neck cancers. The prodrug nanovesicles accomplish favorable tumor accumulation and deep tumor penetration via MMP‐2‐mediated cleavage of the PEG corona, achieving tumor‐specific codelivery of OXA. The OXA prodrug MG‐HOC sensitized tumor cells to ion radiation and efficiently suppressed tumor growth in an SCC7 head and neck mouse model. Additionally, the JAKi remarkably activated tumor‐infiltrating CTLs by abolishing chemoradiotherapy‐induced PD‐L1 expression on the surface of the tumor cells. Consequently, the two‐in‐one prodrug nanovesicles highly efficiently eradicated the HNSCC tumor by self‐cooperatively eliciting an antitumor immune response and migrating acquired immune resistance. The prodrug nanoplatform might represent a novel strategy to enhance chemoradiotherapy of HNSCC and overcome PD‐L1‐dependent immune evasion through the JAK‐STAT pathway.

## Conflict of Interest

The authors declare no conflict of interest.

## Supporting information

Supporting Information

## Data Availability

The data that support the findings of this study are available in the supplementary material of this article.
