# Supplementary material for: Self‐Cooperative Prodrug Nanovesicles Migrate Immune Evasion to Potentiate Chemoradiotherapy in Head and Neck Cancer
Source: Adv Sci (Weinh). 2022 Nov 7;9(36):2203263. doi: 10.1002/advs.202203263 (PMC9798966; doi:10.1002/advs.202203263)
Supplement: Supplementary file 1 — Supporting Information [file ADVS-9-2203263-s001.pdf]

ADVANCED SCIENCE
Open Access

Supporting Information

for *Adv. Sci.*, DOI 10.1002/advs.202203263

Self-Cooperative Prodrug Nanovesicles Migrate Immune Evasion to Potentiate Chemoradiotherapy in Head and Neck Cancer

Yun Zhu, Shunan Zhang, Yi Lai, Jiaxing Pan, Fangmin Chen, Tingting Wang, Fengyang Wang, Zhiai Xu, Wenjun Yang* and Haijun Yu**

Supporting Information

Self-cooperative prodrug nanovesicles migrate immune evasion for potentiated chemoradiotherapy of head and neck cancer

Yun Zhu, Shunan Zhang, Yi Lai*, Jiaxing Pan, Fangmin Chen, Tingting Wang, Fengyang Wang, Zhiai Xu, Wenjun Yang*, Haijun Yu*

Y. Zhu, Prof. W. Yang

Department of Oral and Maxillofacial-Head and Neck Oncology, Ninth People's Hospital, College of Stomatology, Shanghai Jiao Tong University School of Medicine; National Clinical Research Center for Oral Diseases; Shanghai Key Laboratory of Stomatology, Shanghai, 200011, China; E-mail: ywjdoctor@sjtu.edu.cn;

Y. Zhu, S. Zhang, Dr. Y. Lai, F. Chen, J. Pan, Prof. H. Yu

Center of Pharmaceutics, Shanghai Institute of Materia Medica, Chinese Academy of Sciences, Shanghai 201203, China; E-mail: laiyi@simm.ac.cn, hjyu@simm.ac.cn;

Dr. Y. Lai

Department of Gastroenterology, Huadong Hospital, Shanghai Medical College, Fudan University, Shanghai 200040, China;

S. Zhang, Prof. Z. Xu

School of Chemistry and Molecular Engineering, East China Normal University, Shanghai 200241, China;

Dr. T. Wang, Dr. F. Wang

Department of Medical Ultrasound, Shanghai Tenth People's Hospital; Tongji University, Shanghai 200072, China.

Table of Contents

**Experimental section**	Page 36-45
**Figure S1.**	Page 46
**Figure S2.**	Page 47
**Figure S3.**	Page 48
**Figure S4.**	Page 49
**Figure S5.**	Page 50
**Figure S6.**	Page 51
**Figure S7.**	Page 52
**Figure S8.**	Page 53
**Figure S9.**	Page 54
**Figure S10.**	Page 55
**Figure S11.**	Page 56
**Figure S12.**	Page 57
**Figure S13.**	Page 58
**Figure S14.**	Page 59
**Figure S15.**	Page 60
**Figure S16.**	Page 61
**Figure S17.**	Page 62
**Figure S18.**	Page 63
**Figure S19.**	Page 64
**Figure S20.**	Page 65
**Figure S21.**	Page 66
**Figure S22.**	Page 67
**Figure S23.**	Page 68
**Figure S24.**	Page 69
**Figure S25.**	Page 70
**Figure S26.**	Page 71
**Figure S27.**	Page 72

Figure S28. Page 73

Figure S29. Page 74

Figure S30. Page 75

Experimental section

Materials. Fmoc-protected heptapeptide Gly-Pro-Leu-Gly-Leu-Ala-Gly (Fmoc-GPLGLAG) was synthesized by GL Biochem. Co., Ltd (Shanghai, China). Methoxy poly(ethylene glycol) amine ($mPEG_{5k}$-NH_2) was purchased from Seebio Biotech. Co., Ltd (Shanghai, China). Pyropheophorbide a (PPa) was ordered from Dibai Chem-Tech Co, Ltd (Shanghai, China). Oxaliplatin (OXA) was purchased from Platinum Energy. Co., Ltd (Shandong, China). 1,2-dioleoyl-sn-glycero-3-phosphoch-olin (DOPC) was purchased from Advanced Vehicle Technology Pharmaceutical Co., Ltd (Shanghai, China). N-(3-(dimethylamino)-propyl)-N-ethylcarbodiimide hydrochloride (EDCI), 1-hydroxybenzotriazole anhydrous (HOBT), triethylamine (TEA), 4-dimethylaminopyridine (DMAP), and succinic anhydride were all purchased from J&K Scientific Ltd (Beijing, China), Dulbecco's modified eagle medium (DMEM), 0.25% trypsin-EDTA (Phenol Red) and PBS buffer solution (1 ×) were ordered from Meilun Biotech Co., Ltd (Dalian, China). Glutathione (GSH) was purchased from Sigma-Aldrich (Shanghai, China). Cell Counting Kit-8 was purchased from Dalian Meilun Biotech CO., Ltd (Dalian, China).

The mouse lymphocyte separation medium was obtained from Dakewe Biotech (China). Antibodies against CD11c, CD80, CD86, CD45, CD3, CD4, CD8, IFN-γ, CD62L, CD44 and CD274 for flow cytometry were all obtained from BD Biosciences (USA). Antibodies against p-STAT1 for western blot were ordered from CST (USA). Antibodies against JAK2, p-JAK2, STAT1, PD-L1, Actin B, H2AX, Calreticulin, and HMGB1 were ordered from Abcam (UK). IFN-γ, GM-CSF, IL-4, and ELISA kit of

IFN-γ were purchased from Neobioscicence Technology Co., Ltd (USA).

Cell lines and animals. SCC7 murine tumor cells were purchased from the Chinese Academy of Sciences (Shanghai, China). Cells were maintained in DMEM (Gibco) supplemented with 10% fetal bovine serum (FBS), 2 mM of L-glutamine, 1 mM of sodium pyruvate, 0.1 mM of non-essential amino acids, and 1% Penicillin-Streptomycin at 37 °C in 5% CO_2.

For *in vivo* studies, C3H mice (4-5 week old) were obtained from the Shanghai Experimental Animal Center (Shanghai, China). All mice were kept under the pathogen-free condition and used following the experimental animal guidelines approved by the Institute of Animal Care and Use Committee, Shanghai Institute of Materia Medica, Chinese Academy of Sciences.

Bioinformatic analysis. Microarray data and clinical information of HNSCC samples were obtained from The Cancer Genome Atlas (TCGA). Patients with missing or insufficient data were excluded. Finally, 502 tumor samples and 44 normal samples were included. 175 samples had achieved platinum chemotherapy or radiotherapy within 502 tumor samples. Differential expression analysis was conducted on 175 treatment samples and 44 normal samples.

The DEGs were identified by differential expression analysis conducted on R software. 'Limma' package was utilized and the threshold was set as $p < 0.05$ and $|\log(FC)| > 1$. Further functional enrichment analysis was performed and visualized by using 'cluster profile', 'enrichplot', and 'ggplot2' packages in R. Statistical significant terms were finally screened out. The diagrams were generated by the Xiantao web

(https://www.xiantao.love).

Synthesis of PPa-GPLGLAG-PEG and PPa-PEG. MMP-2-sensitive PPa-GPLGLAG-PEG conjugate was synthesized by the following method. Firstly, Fmoc-GPLGLAG (100 mg, 0.12 mmol), EDCI (38.3 mg, 0.2 mmol), HOBT (27.1 mg, 0.2 mmol) and TEA (28 μL, 0.2 mmol) were dissolved in 10 mL of anhydrous DMF. The mixture solution was stirred for about 1.5 h in an ice bath to activate the carboxyl group. Then PEG-NH_2 (0.5 g, 0.1 mmol) dissolved in anhydrous DMF was added to the above mixture solution. After stirring for 24 h at room temperature (RT), the product was purified by dialyzing against ethanol and DI water. After lyophilization, we obtained 519 mg of purified Fmoc-GPLGLAG-PEG as a white powder (yield 89.5%).

Secondly, Fmoc-GPLGLAG-PEG (400 mg, 0.07 mmol) was dissolved in anhydrous DMF with 20% (v/v) 4-Methylpiperidine and stirred overnight at RT to remove the Fmoc group. The reaction mixture was purified by dialysis for two days, followed by lyophilization to obtain NH_2-GPLGLAG-PEG as a white powder (361.8 mg, yield 92.3%).

To synthesize PPa-GPLGLAG-PEG, PPa (25.6 mg, 0.05 mM), EDCI (15.3 mg, 0.08 mM), HOBT (10.8 mg, 0.08 mM) and TEA (11 μL, 0.08 mmol) were dissolved in 3 mL anhydrous DMF. The mixture solution was stirred for about 1.5 h at 0 ℃ to activate the carboxyl group. Then NH_2-GPLGLAG-PEG (224 mg, 0.04 mM) dissolved in anhydrous DMF was slowly added to the above mixture and stirred for 48 h at RT. Subsequently, the solution was dialyzed against DMSO and DI water for two days. PPa-

GPLGLAG-PEG was obtained as a black powder after lyophilization. (220.1 mg, yield 90.2%).

MMP-2 non-responsive PPa-PEG was synthesized in a similar way to PPa-GPLGLAG-PEG. Briefly, PPa (25.6 mg, 0.05 mmol) was activated with EDCI (15.3 mg, 0.08 mmol) and HOBT (10.8 mg, 0.08 mmol) in 3 mL of anhydrous DMF for 1.5 h. Afterward, the mixture was added to 10 2 mL of DMF solution of PEG-NH_2 (200.0 mg, 0.04 mmol) and TEA (11 μL, 0.08 mmol). The reaction was continued in dark at room temperature for 48 h; then, the solution was dialyzed against DMSO and DI water to obtain PPa-PEG as dark powder. (200.9 mg, yield 91.7%).

The chemical structures and relative molecular masses of intermediates and the final product were confirmed using ^{1}H-NMR and Matrix-Assisted Laser Desorption Ionization Time of Flight Mass Spectrometry (MALDI-TOF).

Synthesis of oxaliplatin prodrug hexadecyl-OXA(IV) carboxylic acid (HOC). GSH-sensitive oxaliplatin prodrug was synthesized according to our previously published procedure. Firstly, oxaliplatin (OXA,1 g, 2.5 mmol) was oxidized with 30% H_2O_2, and the suspension was stirred for 24 h at RT. The resulting product was precipitated in cold ethyl ether, washed twice with cold ethyl ether, and dried under vacuum to obtain OXA-OH as a white powder (1006 mg, yield 93.4%). Secondly, OXA-OH (500 mg, 1.16 mmol) and succinic anhydride (116 mg,1.16 mmol) were dissolved in 10 mL DMSO and stirred for 24 h under dark at RT. The reaction solution was precipitated with cold ethyl ether, thoroughly washed with cold ether, and vacuum dried to obtain OXA carboxylic acid (OXA-COOH) as a pale-yellow powder (567.3

mg, yield 92.1%). Finally, hexadecyl isocyanate (360 mg, 1.36 mmol) was added into the solution of OXA-COOH (400 mg, 0.75 mmol) dissolved in 5 mL of DMF. After 24 h reaction in dark at room temperature, the reactant was precipitated and washed with cold ethyl ether. The precipitation was vacuum dried to obtain the final product hexadecyl-OXA carboxylic acid as a white powder (HOC, yield 75.4%). The chemical structures and relative molecular mass of all the intermediates and the final product were characterized by ^{1}H-NMR and electrospray ionization mass spectrometric (ESI-MS) examination.

MMP-2 sensitivity of PPa-GPLGLAG-PEG. To investigate the MMP-2 cleavage activity of PPa-GPLGLAG-PEG, PPa-GPLGLAG-PEG (1.0 mg/mL) and MMP-2 (200 μg/mL) in 20 mM Tris buffer (50 mM NaCl, 100 mM $CaCl_2$, 0.05% Brij35, pH 7.4) were mixed and incubated at 37 °C for 1 h. The process was monitored using HPLC (Waters e2695, C18 column, 5 μm, 4.6 × 250 mm). A linear gradient of methanol/water (from 60%/40% (v/v) methanol/water to 100% methanol in 10 min) was used as the eluent.

Preparation and characterization of prodrug vesicles. To prepare MG-HOC prodrug vesicle, DOPC, PPa-GPLGLAG-PEG, and HOC were mixed with the molar ratio of 76.3:3.3:12.5 and dissolved in the mixed solvent of chloroform and methanol (v/v = 1:9). Then the mixture was vacuum dried to form a homogeneous lipid film. The lipid film was hydrated in PBS (pH 7.4) for 10 min at 48 °C, and extruded through 200 and 100 nm polycarbonate filters to obtain uniform MG-HOC liposomes. Similar procedures were followed to prepare G-HOC by using PPa-PEG instead of PPa-

GPLGLAG-PEG. To prepare MG-HOC@Rux prodrug vesicle, DOPC, PPa-GPLGLAG-PEG, HOC, and Rux were mixed with the molar ratio of 65.4:20.1:2.5:12.0, and dissolved in the mixed solvent of chloroform and methanol (v/v = 1:9).

The hydrodynamic diameter, polydispersity (PDI) of size distribution, and morphology of the G-HOC, MG-HOC, and MG-HOC@Ruxx nanoparticles were examined using dynamic light scattering (DLS) measurement (Zetasizer Nano ZS90, Malvern Instrument, UK) and TEM (Talos L120C, USA, 120 kV).

Cellular uptake of prodrug nanoparticles *in vitro*. SCC7 cells grown in a 24-well plate were incubated with MG-HOC at the desired time points (*i.e.*, 2, 4, 8, and 12h). The intracellular fluorescence intensity of PPa was then examined by flow cytometric measurement and visualized by CLSM to verify the active tumor targeting profile of MMP-2.

Toxicity of the prodrug nanoparticles. CCK-8 assay was performed to evaluate the cell viability. SCC7 cells were seeded and maintained for 24 h in 96-well plates at a density of 8000 cells/well. After incubating with different concentrations of the prodrug nanoparticles (OXA concentration, 1, 2, 5, 10, 20, and 40 μg/mL) for 24 h, the cells were cultured and examined by CCK8 assay.

Western blot assay. The cells were collected and lysed with RIPA lysis buffer containing 1% protease inhibitor. Equal amounts of protein were loaded and separated using SDS-PAGE gels and then transferred to PVDF membranes. Blocking with 5% BSA in TBST for 1h, membranes were conjugated to primary antibodies of p-STAT1 (1:1000), STAT1 (1:300), JAK2 (1:500), p-JAK2 (1:600), PD-L1 (1:300), Actin B

(1:1600) overnight at 4 °C. After incubation with HRP-conjugated secondary antibodies for 1h at room temperature, signal visualization was carried out using a gel imager (Bio-Rad Laboratories, USA) and analyzed by Image J (NIH, USA).

Immunogenic cell death *in vitro*. CRT exposure and nuclear HMGB1 efflux were detected to verify the ICD effect of OXA, MG-HOC, and MG-HOC+RT on SCC7 cells. In brief, cells were seeded on 12-well plates at a density of 2×10^5 cells/well and cultured for 24 h. This was followed by incubating nanoparticles at an OXA concentration of 5.0 μg/mL. The cells were then irradiated with an X-ray at a dose of 8.0 Gy for 24 h. For detecting surface CRT expression, the cells were stained with FITC-conjugated monoclonal secondary antibody for an additional 30 min under dark conditions after incubation with anti-CRT primary antibody for 30 min. The cells were then analyzed by flow cytometry.

To visualize surface CRT and nuclear HMGB1 by immunofluorescent staining, cells were inoculated into 24-well plates with prepared slides and repeated the same experimental conditions. The adherent cells were washed twice with PBS and then fixed with 4% paraformaldehyde for 20 min. To detect HMGB1 efflux, the cells were permeabilized in 0.1% Triton X-100 for 5 min and then blocked in 5% FBS for 1 h at room temperature. The HMGB1 and CRT staining were processed as described above. After staining with DAPI for 5 min, the cells were examined by CLSM (Leica, Germany).

DC maturation *in vitro*. To validate whether the pre-treated tumor cells can promote DC maturation *in vitro*, we extracted the bone marrow-derived monocytes from 6-8

weeks old C3H mice. Monocytes were cultured in complete RPMI containing mouse recombinant GM-CSF (20 ng/mL) and IL-4 (20 ng/mL) for 7 days to differentiate into CD11c$^+$ BMDCs. BMDCs were stimulated with treated tumor cells for 24 h, then stained with fluorescence-labeled antibodies against CD11c, CD80, and CD86. CD80/CD86 double-positive DCs were recognized as maturated DCs and were gated from CD11c$^+$ DCs.

Tumor targeting and biodistribution of the prodrug nanoparticles *in vivo*. SCC7 tumor-bearing mice were intravenously injected with PPa-loaded nanoparticles at a 2.0 mg/kg dose. Biodistribution of nanoparticles *in vivo* at the different time points (2, 4, 8, 12, and 24 h) was determined by IVIS Lumina II In Vivo Imaging System (Caliper LifeSciences)) (Ex/Em = 670 nm/720 nm). The major organs (heart, liver, spleen, lung, and kidney) and tumors were collected and likewise imaged *ex vivo* 24 h post-administration.

Antitumor study and biosafety assay *in vivo*. To explore the antitumor effect of the nanoparticle system, the SCC7 tumor-bearing C3H mice model was established by subcutaneous injection of 2×10^6 SCC7 cells on the right flank of mice. When the tumor volume reached 200 mm^3, the mice were randomly divided into the following groups (n = 5): PBS, OXA, OXA+RT, RT, MG-HOC, and MG-HOC+RT. The mice have intravenously injected with nanoparticles at an equal dose of Oxa (1.0 mg/kg). Twenty-four hours later, the tumors in RT groups were locally irradiated with X-ray at a dose of 4.0 Gy. The intervention was repeated three times every two days. The tumor size and body weight were recorded every other day during the observation period. The

tumor volume was determined following the formula:

Tumor volume = length × width × width/2 (length, the longest dimension; width, the shortest dimension).

At the endpoint of the antitumor study, the major organs (heart, liver, spleen, lung, and kidney) and tumors were preserved for HE or TUNEL staining to examine biosafety and apoptosis of tumor cells.

Immune assay *in vivo*. To further investigate the induction of the protective antitumor immune effect, when the tumor volume reached 400 mm^3, the mice were randomly divided into four groups (n = 5): PBS, MG-HOC+RT, MG-HOC@Rux, and MG-HOC@Rux+RT. The mice were intravenously injected with nanoparticles at an equal dose of OXA (1.0 mg/kg) and Rux (2.0 mg/kg). Twenty-four hours later, the tumors in RT groups were locally irradiated with X-ray at a dose of 4.0 Gy. The intervention was repeated three times every two days. Tumors and lymph nodes were harvested 7-days post the definitive treatment. Briefly, tumors were cut into small pieces after weighing, then suspended in RPMI160 with indicated digestive enzymes and dissociated by the gentle MASC^{TM} dissociator (Miltenyi, German). The single-cell suspension was obtained by filtering through 70 μm filters. Tumor-infiltrating lymphocytes (TILs) were separated and enriched by a mouse lymphocyte separation medium. After counting, the TIL suspension was stained with anti-CD45-APC, anti-CD3-FITC and anti-CD8-PE to quantify the subpopulations of $CD8^+$ T cells. For cytotoxic lymphocyte subsets, i.e. (CTLs, IFN-$\gamma^+$$CD8^+$ T cells), the single-cell suspension was pre-treated with permeabilization and stained with anti-CD3-FITC, anti-CD8-PE, and anti-IFN-γ-

AF647 antibodies. All samples were incubated with specific antibodies for 30 min at 4 °C in the dark, then measured by FACS fortessa flow cytometry (Bioscience) and analyzed with FlowJoV10 software. Data were presented after normalization according to the tumor weight and lymphocytes counting.

To determine DC maturation *in vivo*, lymph nodes were ground into a single cell suspension and stained with anti-CD11c-FITC and anti-CD80-PE. Matured DCs ($CD11c^{+}CD80^{+}$) were then analyzed by flow cytometry.

To investigate the memory T lymphocytes in spleens post-treatments, the single-cell suspension of spleens was collected by filtering after gentle grinding and purified by erythrocyte lysis buffer. Memory T cells ($CD8^{+}CD44^{+}CD62L^{-}$) stained with anti-CD8-FITC, anti-CD44-APC, and anti-CD62L-PE were detected using flow cytometry.

Statistical analysis. All experiments were carried out for at least three replicates. Data were presented as mean ± standard deviation (SD). Statistical differences were calculated by two-tailed Student's t-test between two groups and one or two-way ANOVA among multiply groups performed in Graphpad Prism 9.0. $p < 0.05$ was considered statistically significant.

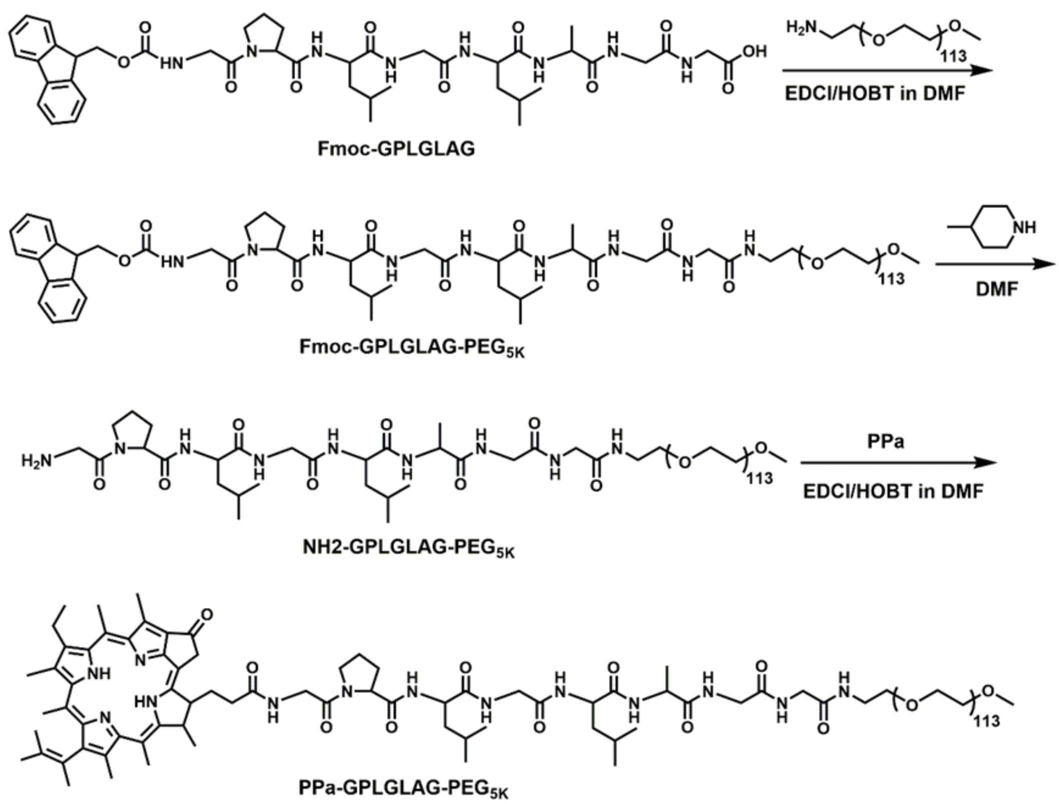

Figure S1. The **s**ynthetic route of PPa-GPLGLAG-PEG.

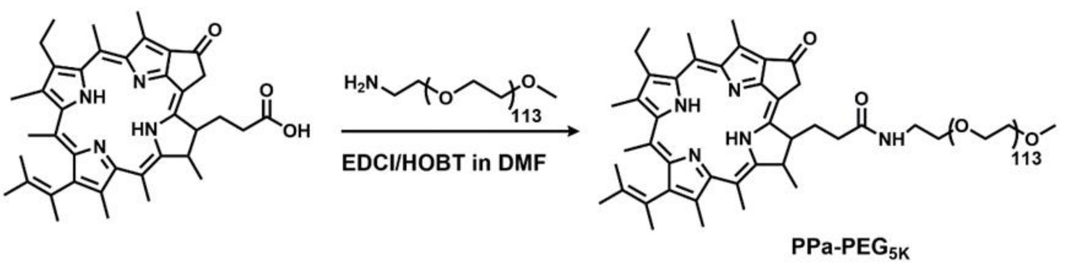

Figure S2. The **s**ynthetic route of PPa-PEG.

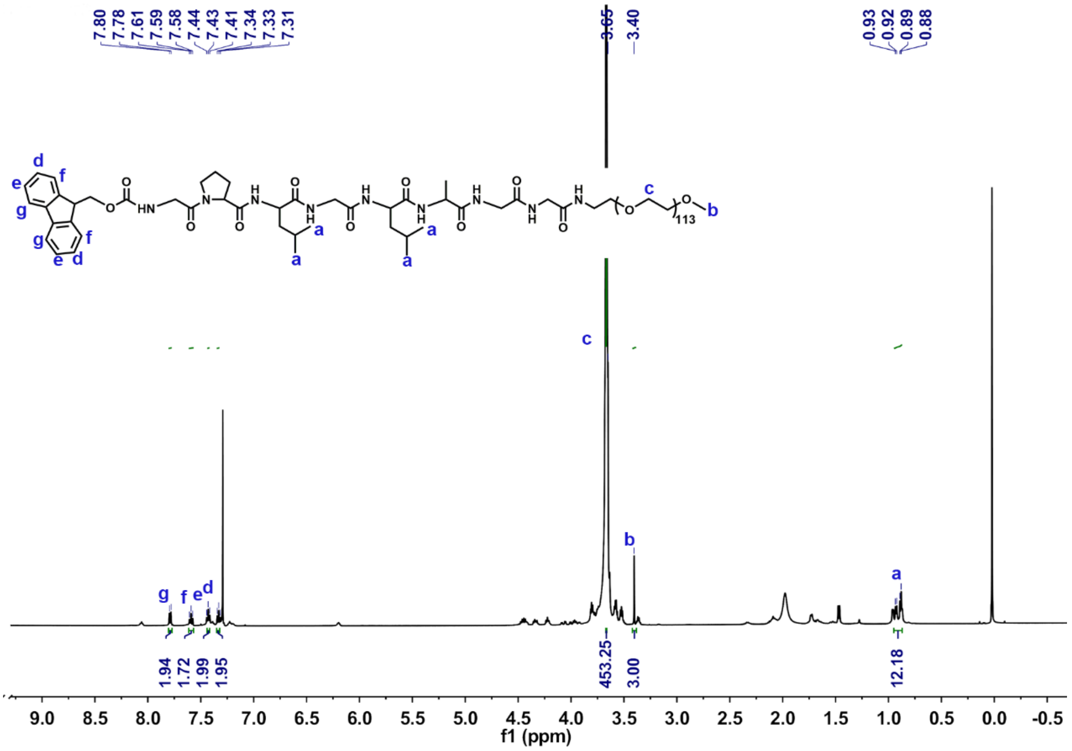

Figure S3. ^{1}H-NMR spectrum of Fmoc-GPLGLAG-PEG.

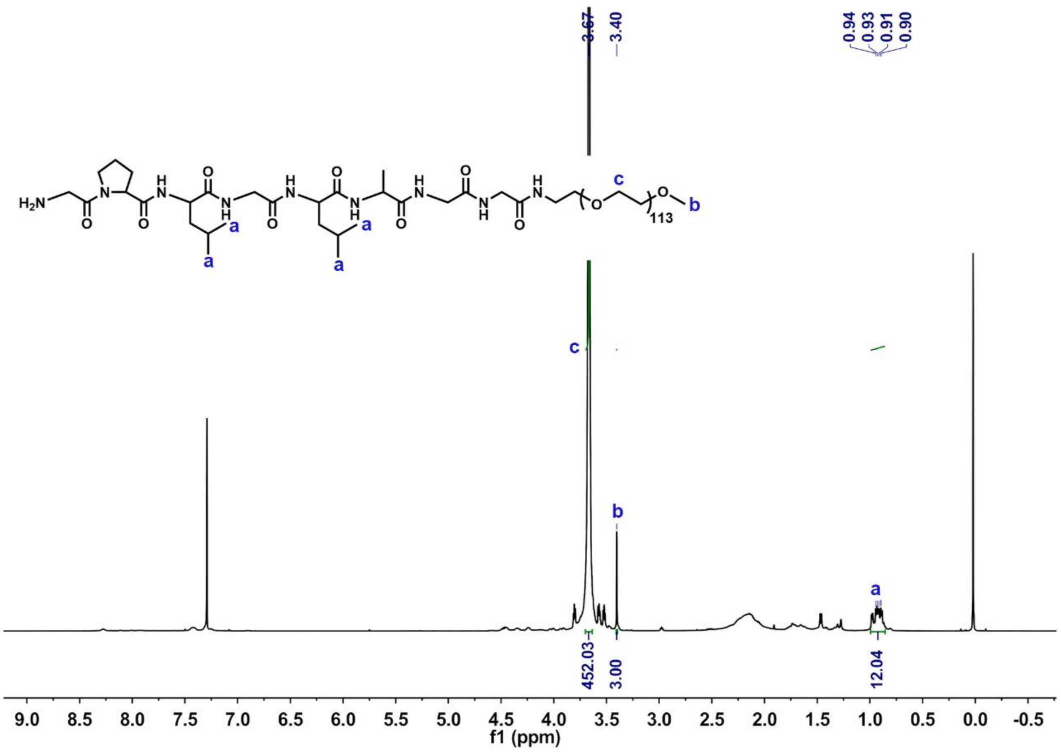

Figure S4. ^{1}H-NMR spectrum of NH_2-GPLGLAG-PEG.

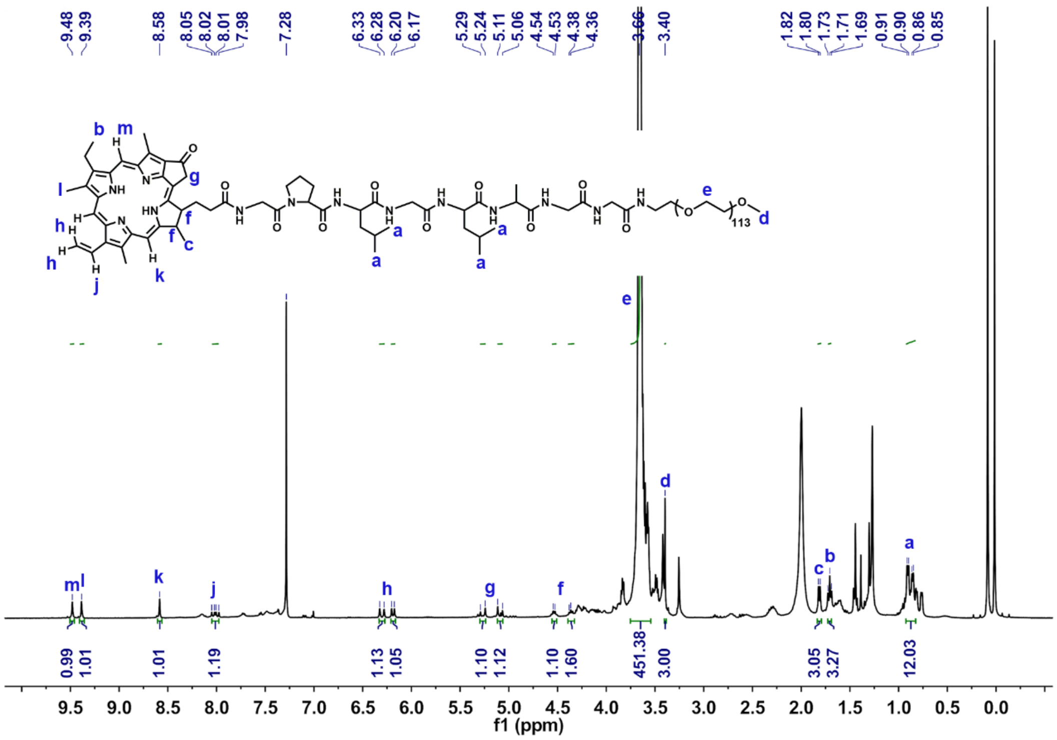

Figure S5. ^{1}H-NMR spectrum of PPa-GPLGLAG-PEG.

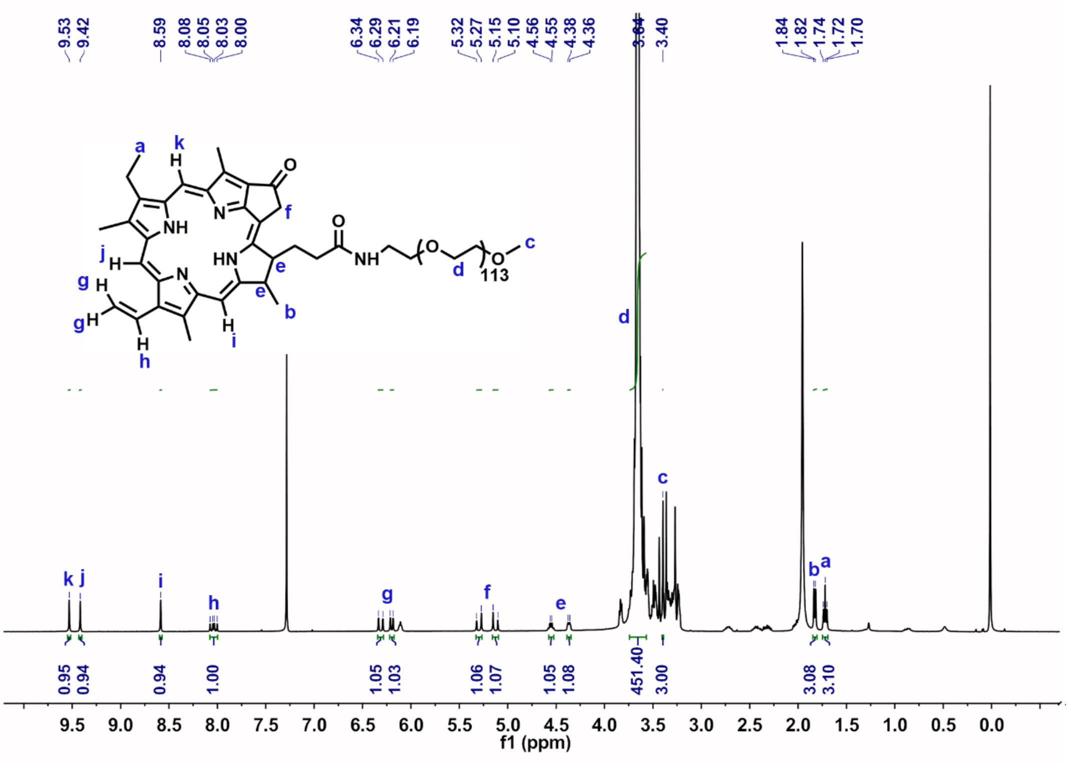

Figure S6. ^{1}H-NMR spectrum of PPa-PEG.

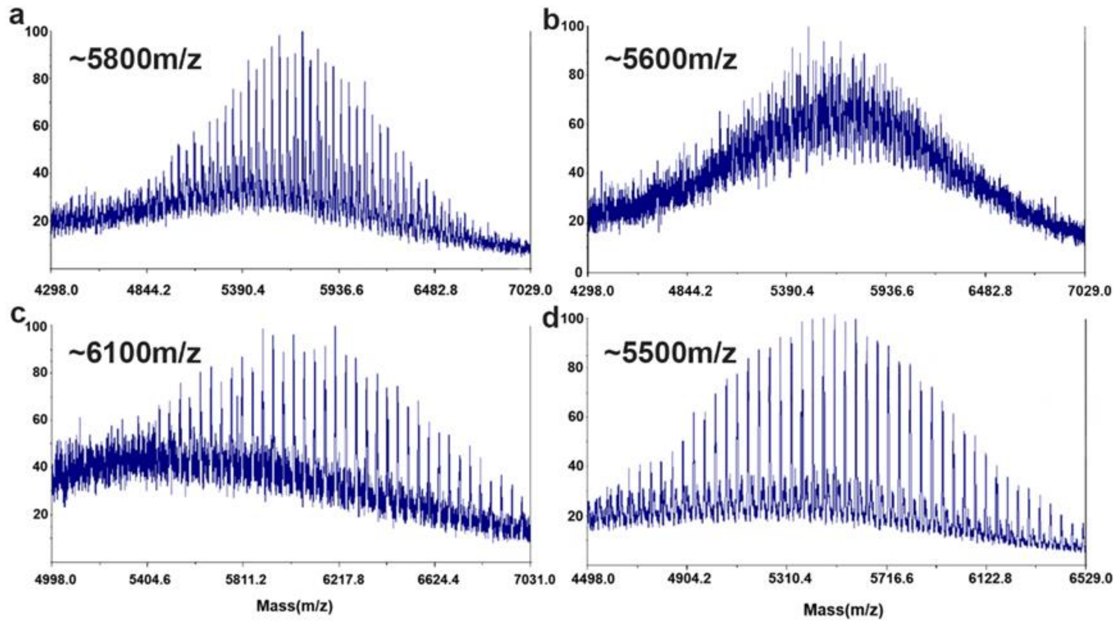

Figure S7. MALDI-TOF MS spectra of (a) Fmoc-GPLGLAG-PEG; (b)NH_2-GPLGLAG-PEG; (c) PPa-GPLGLAG-PEG and (d) PPa-PEG.

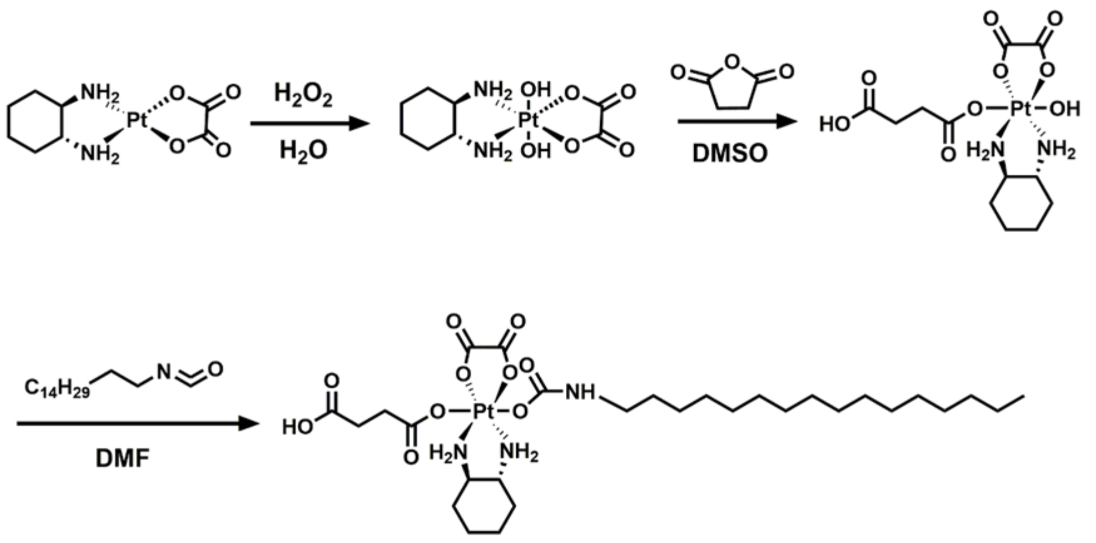

Figure S8. The synthetic route of oxaliplatin prodrug hexadecyl-OXA(IV) carboxylic acid (HOC).

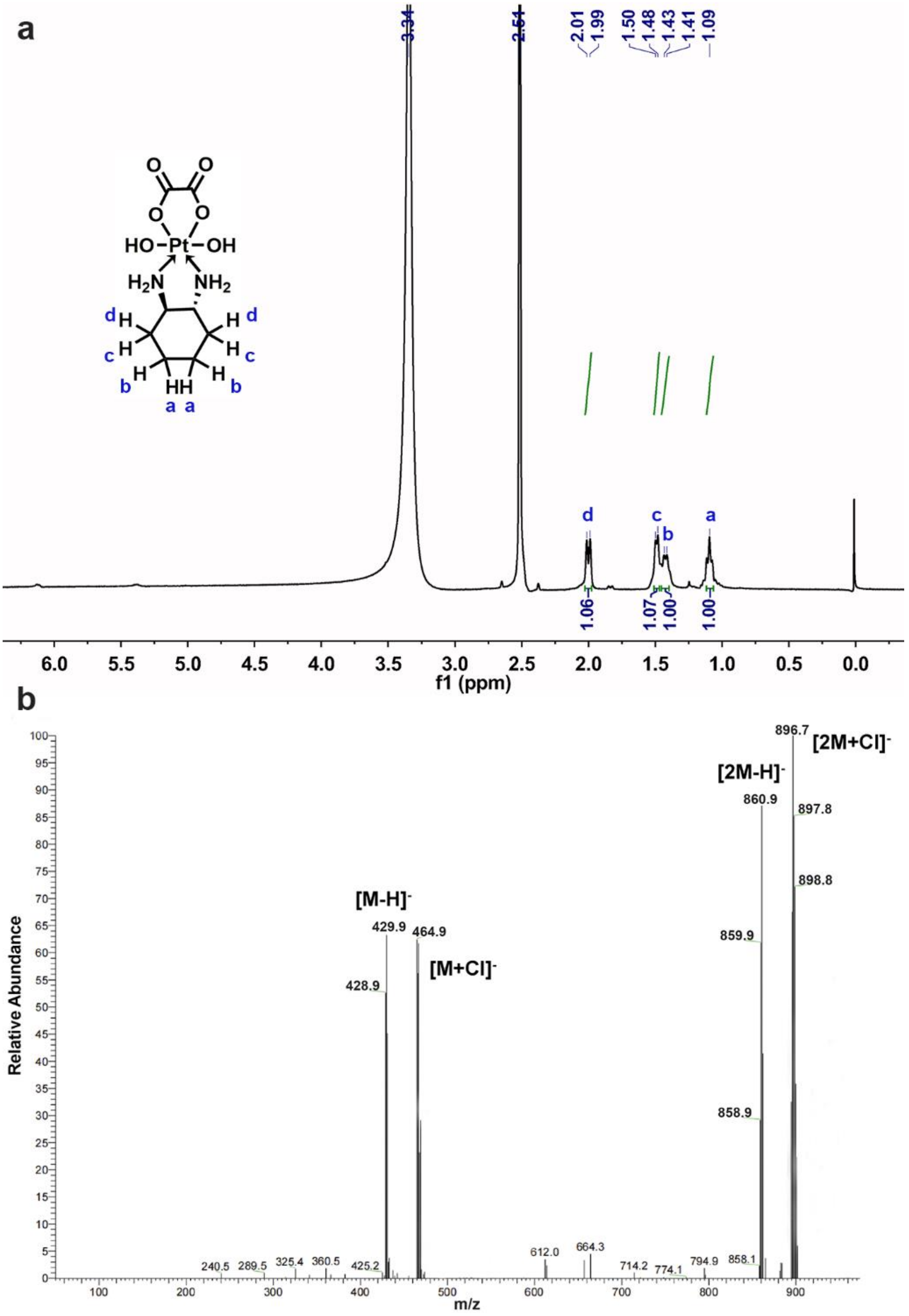

Figure S9. (a) ^{1}H-NMR spectrum and (b) ESI-MS spectrum of OXA-OH.

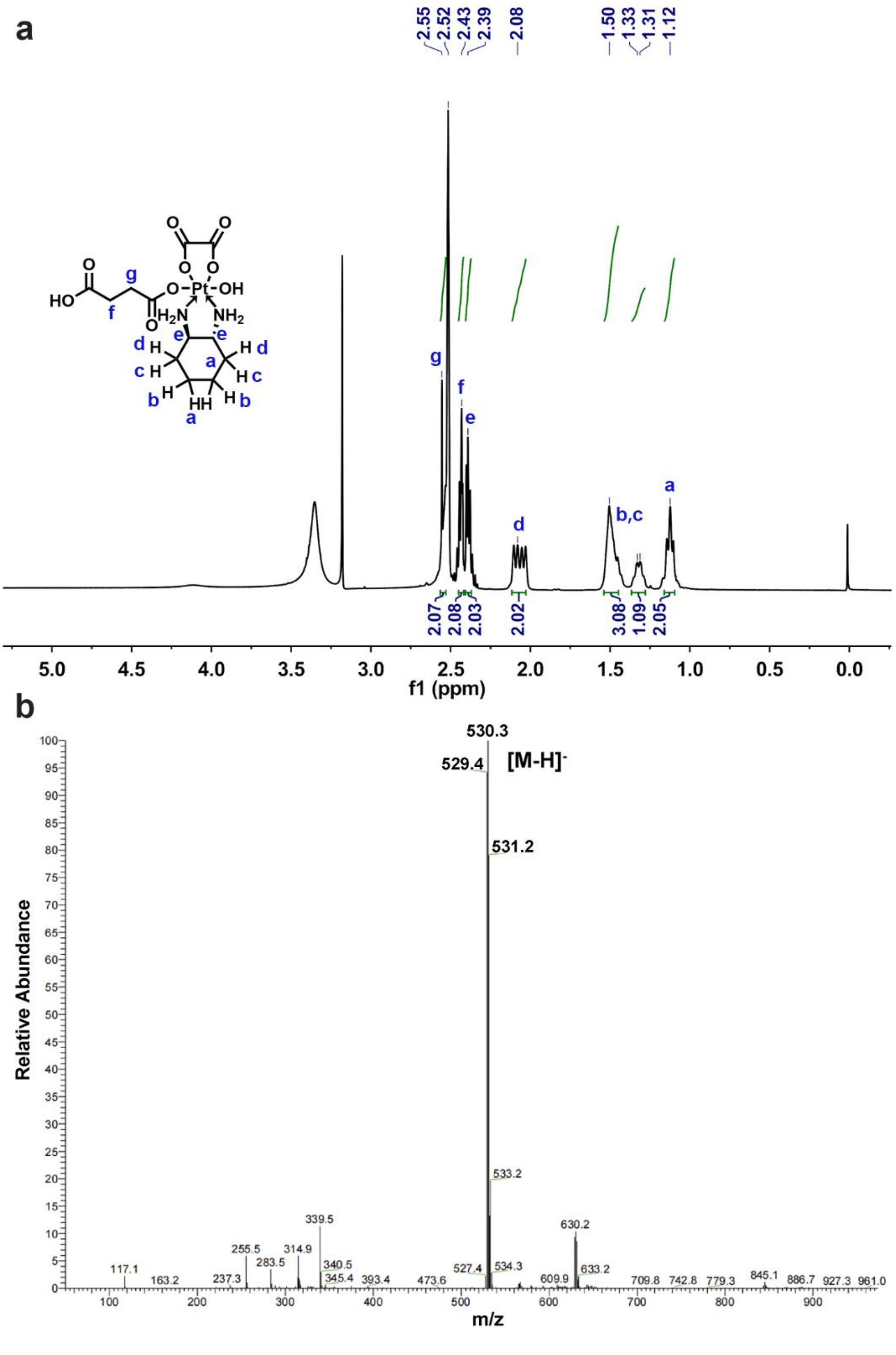

Figure S10. (a) ^{1}H-NMR spectrum and (b) ESI-MS spectrum of OXA-COOH.

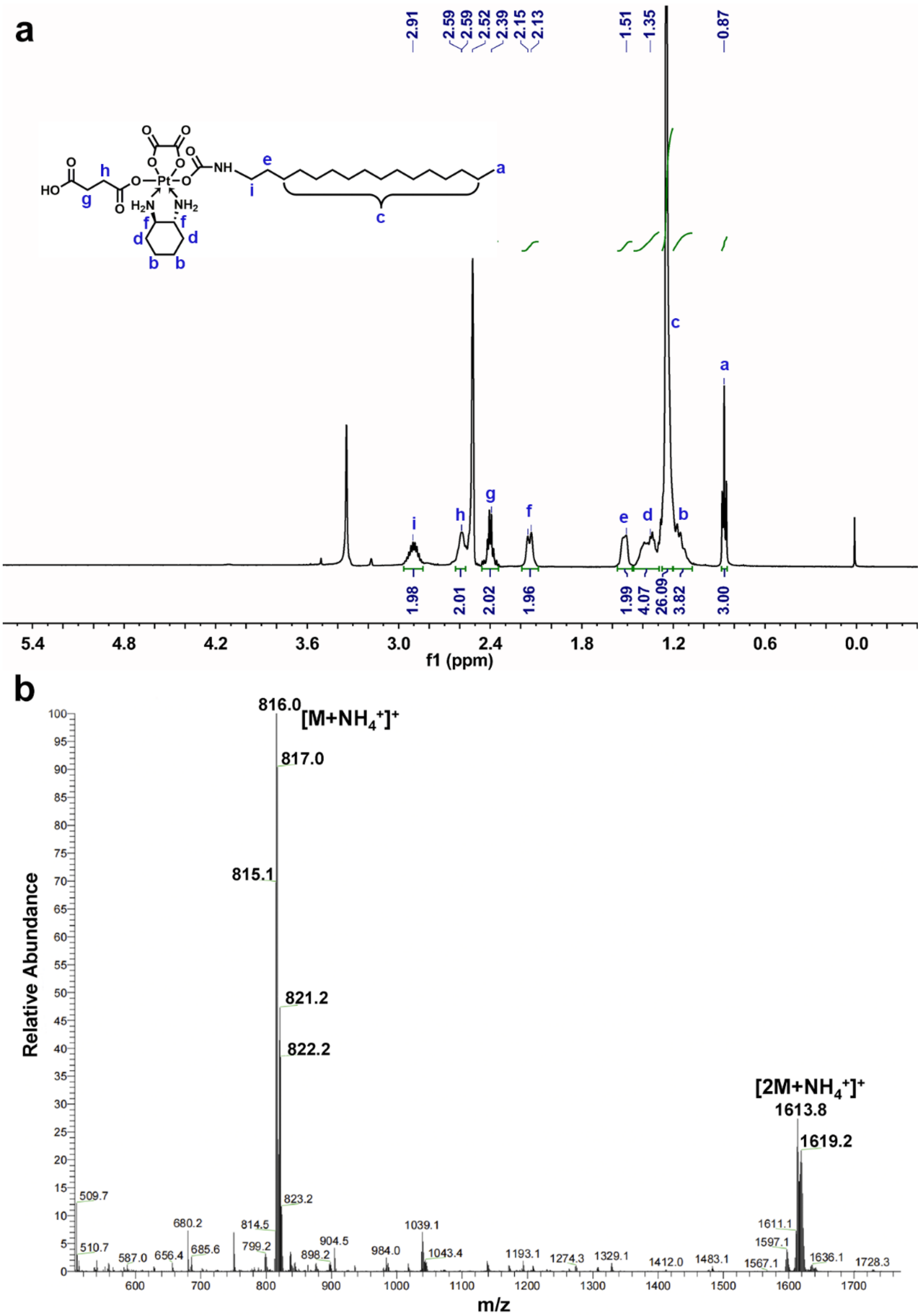

Figure S11. (a) ^{1}H-NMR spectrum and (b) ESI-MS spectrum of HOC.

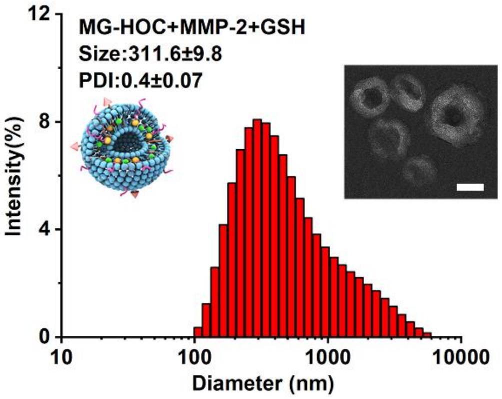

Figure S12. Morphology and particle size distribution of MG-HOC nanovesicles in the presence of MMP-2 and GSH (Insert: TEM images of MG-HOC after 24 h of incubation with 200 μg/mL MMP-2 and 10 mM GSH, scale bar = 200 μm).

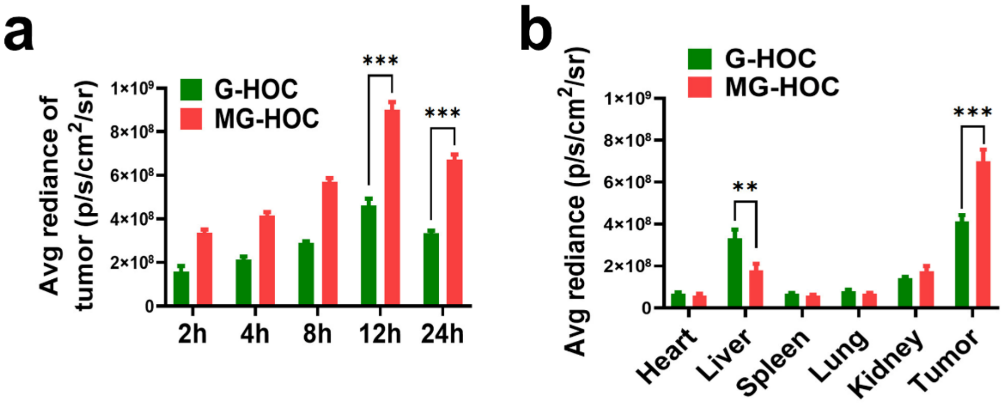

Figure S13. a) Normalized fluorescence intensity of the tumor sites examined 24 h post-injection; b) Normalized fluorescence intensity of the major organs; The data were showed as mean ± SD. *P < 0.05; **P < 0.01; ***P < 0.001; ****P < 0.0001.

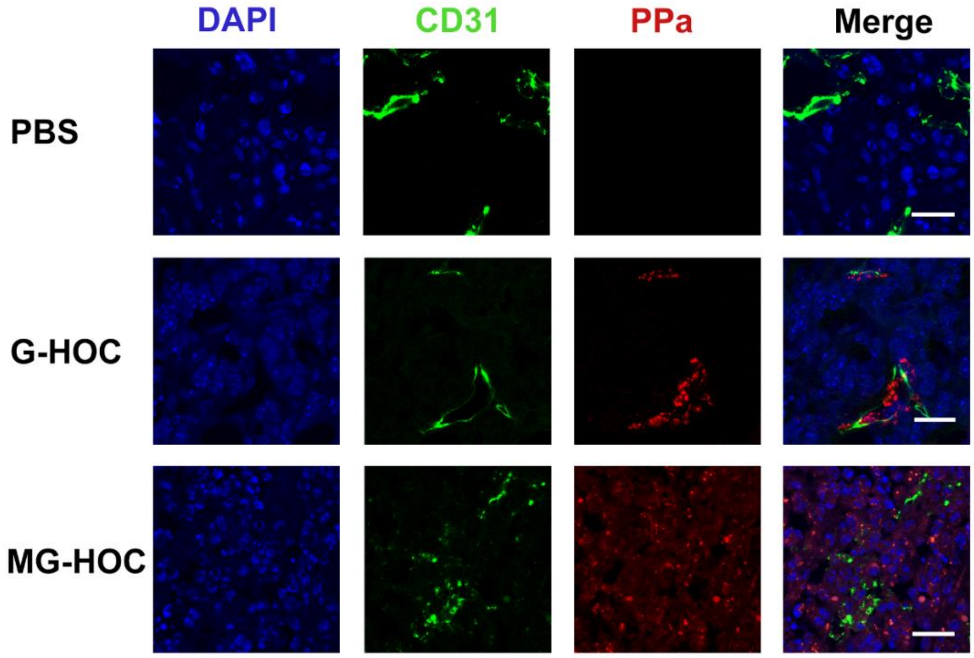

Figure S14. CLSM examination of MG-HOC distribution in SCC7 tumor sections examined 24 h post-injection. SCC7 tumor-bearing mice were intravenously injected with PPa-loaded nanoparticles at a dose of 2 mg/kg. Twenty-four h later, the tumors were collected to prepare the frozen sections and further stained with CD31-antibody (scale bar = 20 μm).

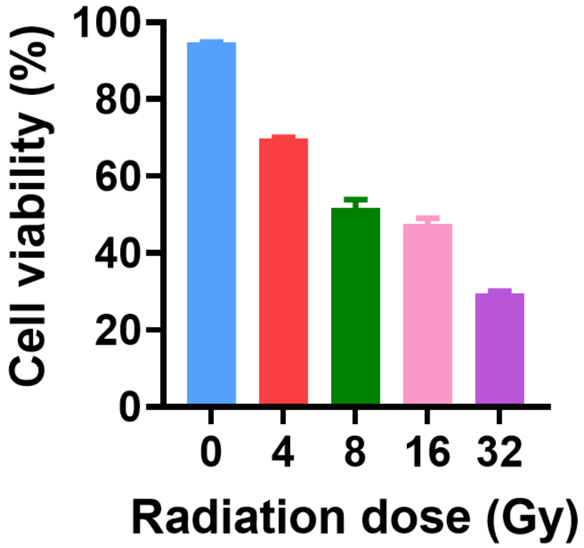

Figure S15. CCk-8 assay-determined cell viability of SCC7 cells after treatment with different doses of X-ray radiation.

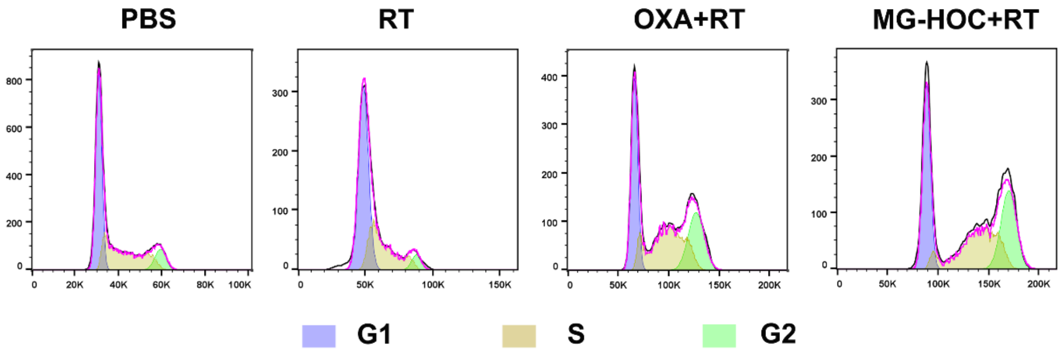

Figure S16. Flow cytometry examination of chemoradiotherapy-induced cell cycle arrest in the G2 phase in SCC7 cells.

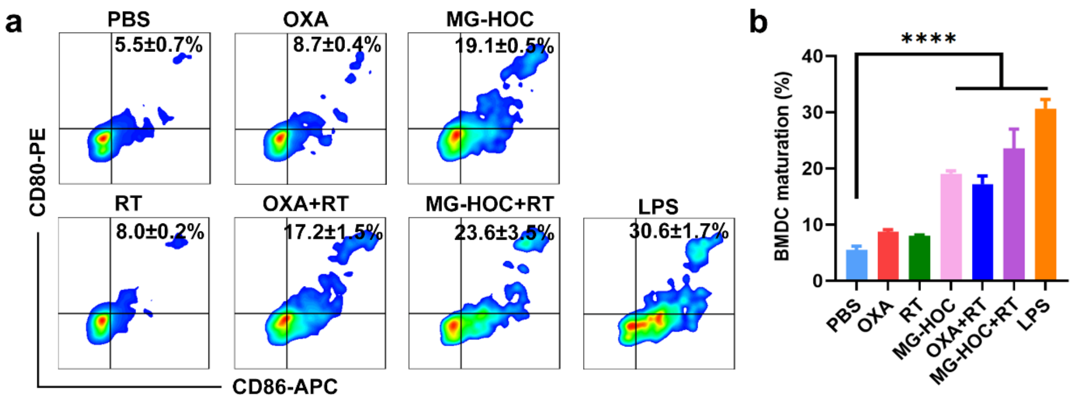

Figure S17. Flow cytometry examination of DC maturation after co-incubation with OXA or prodrug nanoparticles-pretreated SCC7 cells at an OXA concentration of 5 μg/mL and a dose of 8.0 Gy X-ray. a) Representative flow cytometric plots. The tumor cells treated with 1.0 μg/mL of LPS were used as the positive control. b) Averaged BMDC maturation ratio (gated on $CD11c^+CD80^+CD86^+$) stimulated by treated SCC7 cells *in vitro* (n = 3).

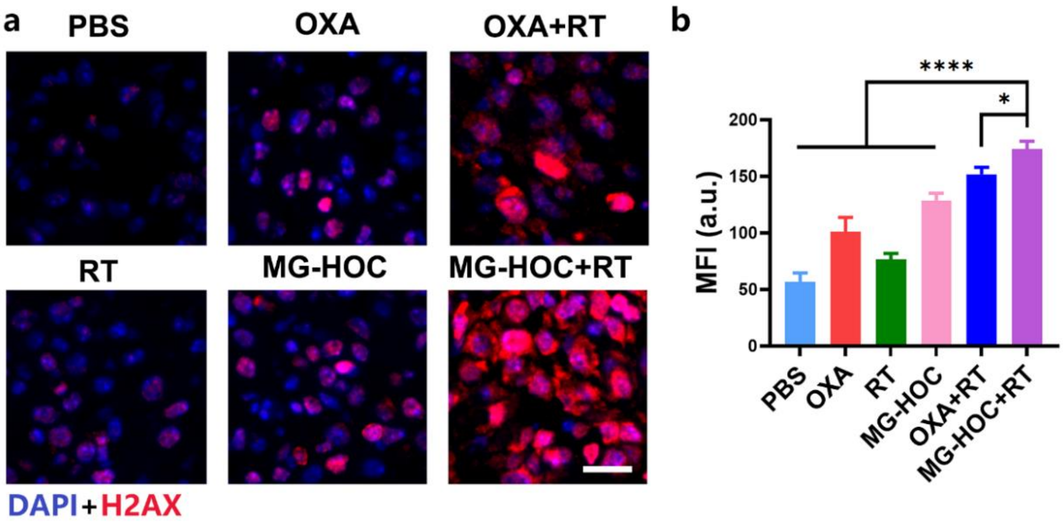

Figure S18. CLSM examination of H2AX expression in SCC7 tumor tissues (scale bar = 50 μm).

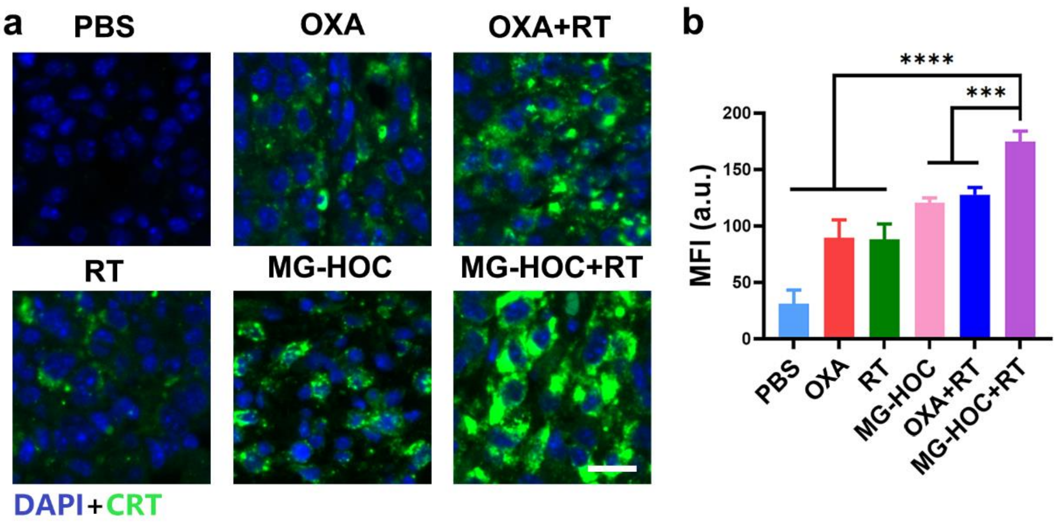

Figure S19. CLSM examination of CRT translocation in SCC7 tumor tissues (scale bar = 50 μm).

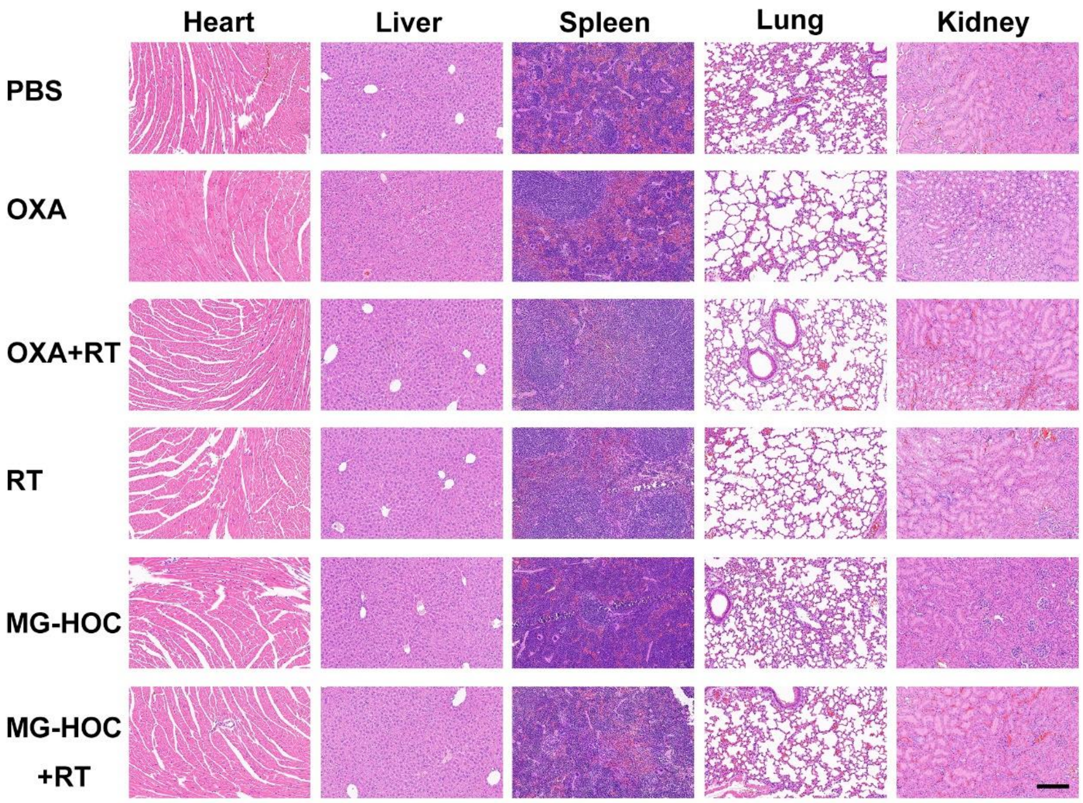

Figure S20. H&E staining of the heart, liver, spleen, lung, and kidney of the SCC7 tumor-bearing mice was examined at the end of the antitumor study (scale bar = 200 μm).

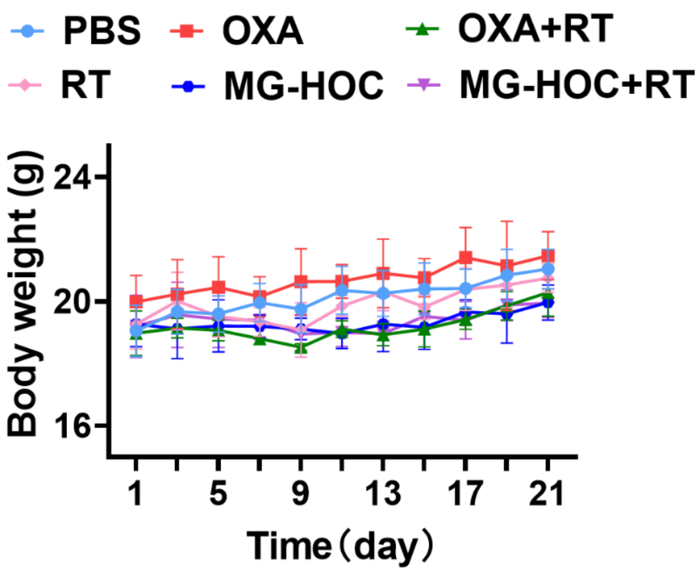

Figure S21. Body weight change of SCC7 tumor-bearing mice examined during the antitumor study (n = 5).

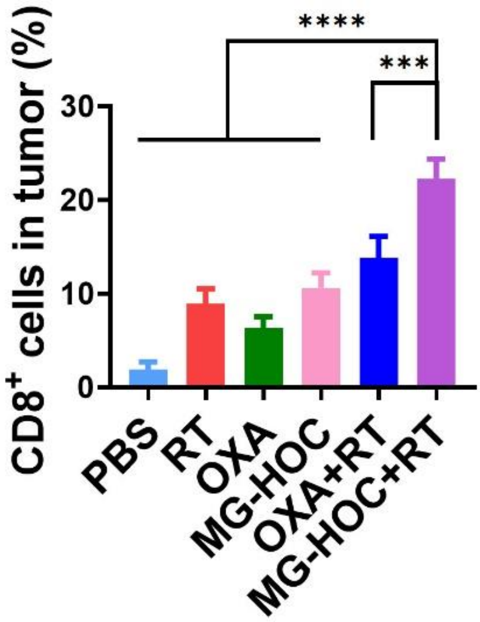

Figure S22. Semi-quantitative analysis of the tumor-infiltrating $CD8^+$ lymphocytes in the SCC7 tumor sections *ex vivo*.

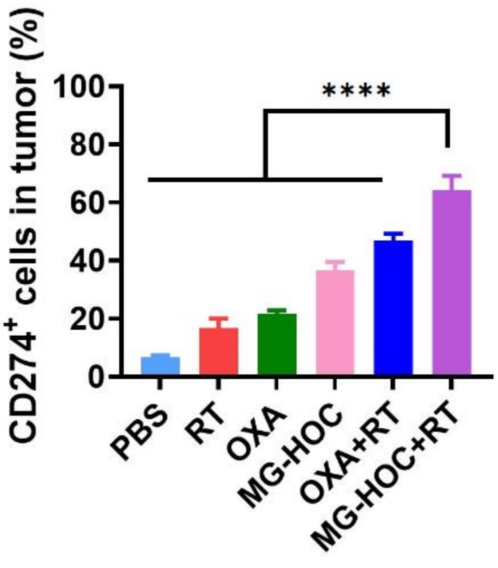

Figure S23. Semi-quantitative analysis of CD274 expression in the SCC7 tumor sections ex vivo.

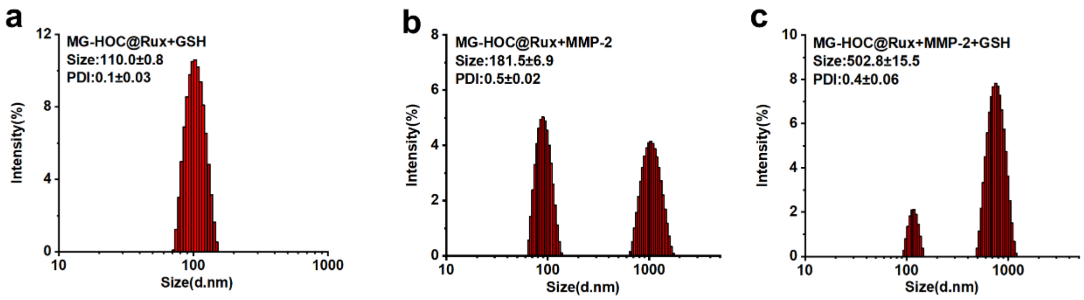

Figure S24. Representative DLS data of the MG-HOC@Rux nanovesicles post 24 h incubation with (a) 10 mM GSH; (b) 200 μg/mL MMP-2; (c) 200 μg/mL MMP-2 and 10 mM GSH.

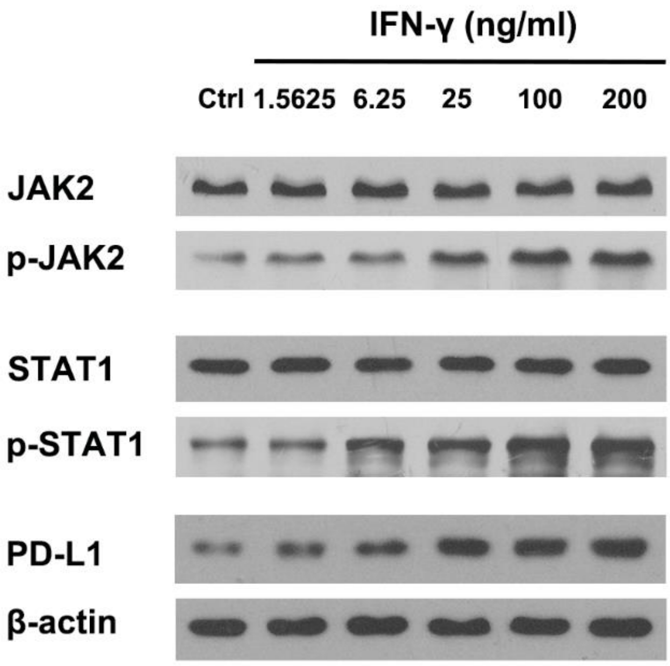

Figure S25. Western-blot assay of PD-L1 expression in SCC7 tumor cells *in vitro*. Examined after 24 h incubation with different concentrations of IFN-γ.

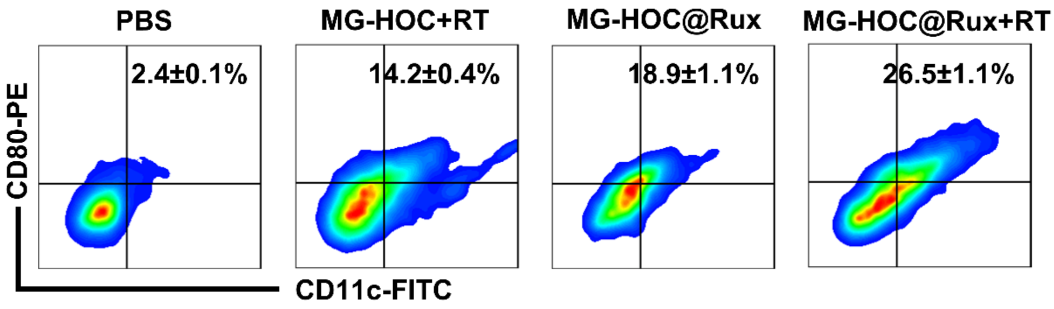

Figure S26. Flow cytometry plots of matured DCs (gated on CD11c$^+$) in the tumor-draining lymph nodes of SCC7 tumor-bearing C3H mice receiving different treatments (n = 3). Flow cytometry was performed 5-days post the indicated treatments.

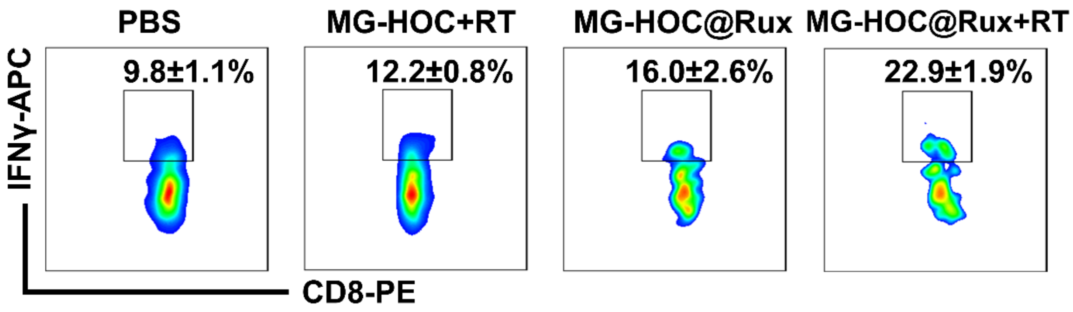

Figure S27. Flow cytometric plots of tumor-infiltrating IFN-γ^+CD8 T cells ($CD3^+CD8^+IFN\text{-}\gamma^+$) in SCC7 tumor-bearing C3H mice receiving different treatments (n = 3). IFN-γ^+CD8 T cells marked by IFN-γ^+CD8+ were determined by flow cytometry at 5-days post the indicated treatments.

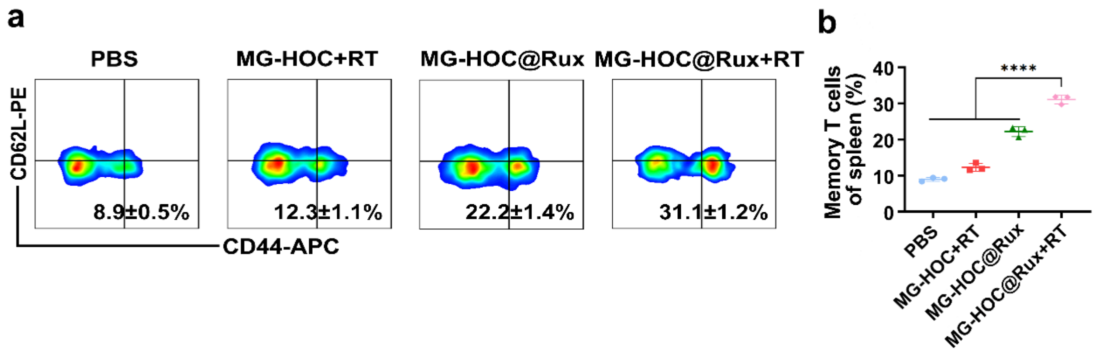

Figure S28. a) Flow cytometry analysis, and b) quantification of memory T lymphocytes ($CD8^{+}CD44^{+}CD62L^{-}$) in the spleen of SCC7 tumor-bearing C3H mice examined 5-days post the indicated treatment (n = 3).

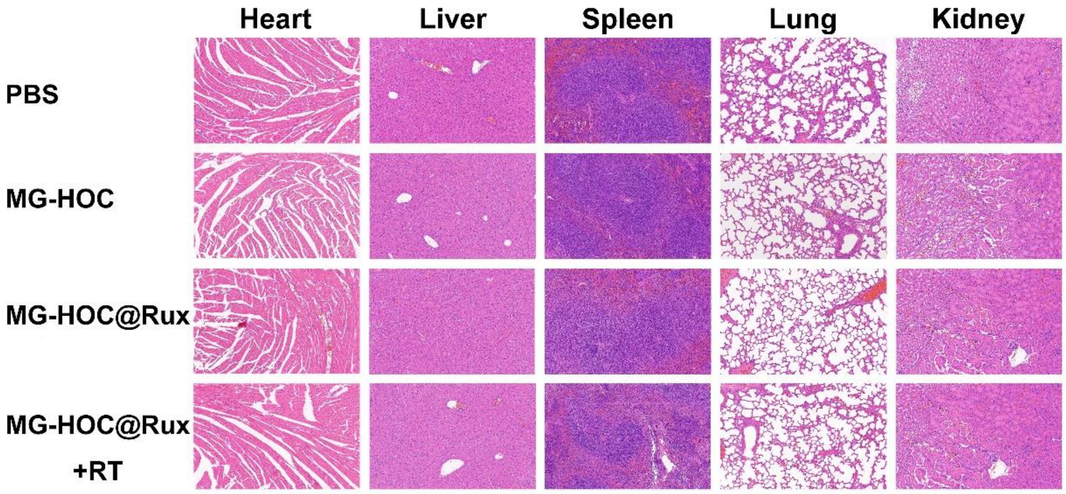

Figure S29. H&E staining of the heart, liver, spleen, lung, and kidney of the SCC7 tumor-bearing mice was examined at the end of the antitumor study (scale bar = 200 μm).

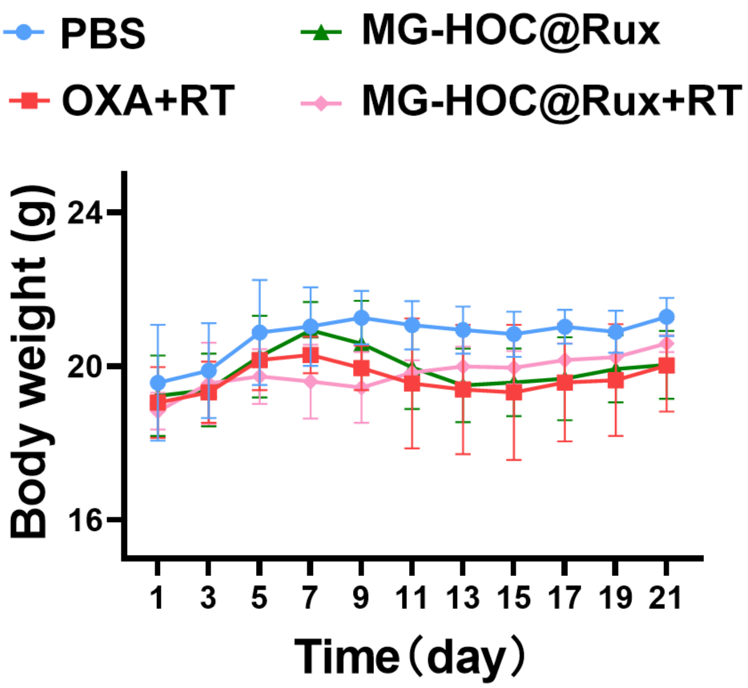

Figure S30. Body weight change of the SCC7 tumor-bearing C3H mice was recorded during the advanced antitumor study.
